# Structural insights into SARS-CoV-2 spike protein and its natural mutants found in Mexican population

**DOI:** 10.1038/s41598-021-84053-8

**Published:** 2021-02-25

**Authors:** Yudibeth Sixto-López, José Correa-Basurto, Martiniano Bello, Bruno Landeros-Rivera, Jose Antonio Garzón-Tiznado, Sarita Montaño

**Affiliations:** 1 Laboratorio de Diseño y Desarrollo de Nuevos Fármacos e Innovación Biotecnológica (Laboratory for the Design and Development of New Drugs and Biotechnological Innovation), Sección de Estudios de Posgrado e Investigación, Escuela Superior de Medicina, Instituto Politécnico Nacional, Plan de San Luis y Salvador Díaz Mirón S/N, Casco de Santo Tomás, 11340 Mexico, Mexico; 2 CNRS, Laboratoire de Chimie Théorique, LCT, Sorbonne Université, Paris, France; 3 Laboratorio de Bioinformática y Simulación Molecular, Facultad de Ciencias Químico Biológicas, Universidad Autónoma de Sinaloa, Culiacán, Sinaloa Mexico

**Keywords:** Computational biology and bioinformatics, Drug discovery

## Abstract

The severe acute respiratory syndrome coronavirus 2 (SARS-CoV-2) is a newly emerged coronavirus responsible for coronavirus disease 2019 (COVID-19); it become a pandemic since March 2020. To date, there have been described three lineages of SARS-CoV-2 circulating worldwide, two of them are found among Mexican population, within these, we observed three mutations of spike (S) protein located at amino acids H49Y, D614G, and T573I. To understand if these mutations could affect the structural behavior of S protein of SARS-CoV-2, as well as the binding with S protein inhibitors (cepharanthine, nelfinavir, and hydroxychloroquine), molecular dynamic simulations and molecular docking were employed. It was found that these punctual mutations affect considerably the structural behavior of the S protein compared to wild type, which also affect the binding of its inhibitors into their respective binding site. Thus, further experimental studies are needed to explore if these affectations have an impact on drug-S protein binding and its possible clinical effect.

## Introduction

The severe acute respiratory syndrome coronavirus 2 (SARS-CoV-2) is a newly emerged coronavirus responsible for COVID-19; it became a pandemic since March 2020. As of August 6, 2020 more than 18,902,735 million people have been infected, and more than 709,511 have died from it worldwide. In Mexico, there are more than 1,241,436 diagnoses cases and more than 113,700 deads, according to the World Health Organization (WHO, accessed on December 14, 2020). The coronavirus entry into the host cell is mediated by the transmembrane spike (S) glycoprotein. Homotrimers of S proteins are surface-exposed and are responsible for the virus attachment to the host receptor located in different human organs, which turns them into the main targets of neutralizing antibodies^1, 2^. The fusion capacity of the S protein is a leading indicator of the infectivity of the virus. The S protein consist of two functional subunits; receptor-binding (S_1_) subunit and fusion viral (S_2_) subunit^2, 3^. S_1_ comprises the receptor-binding domain (RBD) that recognizes angiotensin-converting enzyme-2 (ACE2) as its receptor^4, 5^ and it contributes to the stabilization of the perfusion state of the membrane-anchored S_2_ subunit, which contains the fusion machinery. The S1/S2 processing sites exhibit different motifs among coronaviruses^6^. The priming process is ensured by different host cell proteases depending on the S1/S2 sequence cleavage site. In the case of the SARS-CoV-2, S protein contains a canonical furan-like cleavage site: 681–PRRAR↓SV-688, which is absent in SARS-CoV and others SARS-related coronaviruses^2, 3, 6, 7^, this new site could provide a gain of function that may efficient the virus spreading in the human population compared to other lineages of beta-coronaviruses^6^. Recent studies show SARS-CoV-2 mutating during the continuous transmissions among the population^8, 9^. To date, two of the three reported viral lineages are circulating among Mexican population^10^. The principal SARS-CoV-2 mutations detected worldwide are found within the viral S protein^9, 11–14^, which is critical for the virus attachment to the host cell^11^. However, the S protein’s mutation effects are poorly understood, and these mutations possibly impact the 3D structure and functional behavior. These variations can be used to identify epitopes as targets for vaccine development, as well as antibody and antiviral drug design, to find treatments against SARS-CoV-2^9, 14^.

Since the beginning of the COVID-19 pandemic, several strategies to face the virus have been engaged. One of them consists of drug repurposing. With this strategy, the emerge of marketed drugs with therapeutic potential for COVID-19 treatment has been suggested. Such is the case of cepharanthine, nelfinavir, and hydroxychloroquine. Cepharanthine is a biscoclaurine alkaloid, currently used for radiation-induced leukopenia management, alopecia, idiopathic thrombocytopenic purpura, middle ear catarrh. It possesses anti-inflammatory, antioxidative, immunomodulating, and antiviral properties^15^. Independent studies have demonstrated the potential use of cepharanthine for SARS-COV-2 treatment due to their potent antiviral activity^16–18^, which interferes with the Spike-ACE2 binding evading the viral entry to the host cells^19, 20^. Hydroxychloroquine is used to treat malaria, rheumatoid arthritis, chronic discoid lupus erythematosus, and systemic lupus erythematosus^21^. In vitro antiviral properties against SARS-CoV-2 of hydroxychloroquine attract the attention, suggesting a possible role as a treatment for SARS-CoV-2^22^. In this regard, hydroxychloroquine in vitro inhibitory activity was proven against SARS-CoV-2, with different mechanisms of action, such as sialic acid receptors blockade, cytokine storm prevention, endosomal pH elevation, and ACE-2 terminal glycosylation affectation leading to virus binding evasion^23–25^. Currently, hydroxychloroquine is still being administered for COVID-19 treatment worldwide, but the clinical evidence of its therapeutic effectivity is still emerging; there is contradictory clinical evidence, some studies support the therapeutic efficacy while in others no significant improvements were observed^26, 27^. Therefore, several studies are still being conducted at different levels for explaining hydroxychloroquine activity, one approach is the use of in silico studies, among them, the possible interaction of Hydroxychloroquine with Spike protein, specifically with RBD domain was suggested^20, 28, 29^. Nelfinavir is currently used as an anti-HIV drug with protease inhibitor activity, several studies suggest that it inhibits SARS-CoV replication^30, 31^. Further, Musarrat et al., reported that it suppresses SARS-CoV-2 spike-mediated cell–cell fusion at micromolar ranges, besides molecular docking suggested that it might interact with HR1 region^32^.

The review and comparative assessment of sequences of S protein among available SARS-CoV-2 genomes in the GISAID database showed three major mutations (H49Y, D614G, and T573I) circulating among Mexican population. Multiple crystallized 3D structures of S protein can be found in the Protein Data Bank (PDB)^2, 15–17^. However, to capture conformational changes in the 3D atomistic model, it is necessary to perform molecular dynamics (MD) simulations^33^. To determine if the mutated S proteins found in Mexican population (H49Y, D614G, and T573I) could affect its 3D structure conformations, here we employed MD simulations for full-length atomistic models of S protein 3D structure and its mutants. The most populated cluster conformations of S protein and its mutants, retrieved form MD simulations were further docked with reported S protein inhibitors (cepharanthine, hydroxychloroquine, and nelfinavir)^20, 27, 28^ to explore how the binding affinity and free binding energy was affected due to the conformational changes caused by punctual mutations on S protein and then the structural and energetic stability of the complexes were studied by MD simulation. The in silico results provide evidence of affectation in the binding affinity and the free energy binding values for the three different compounds on the S protein wild type (WT) and its mutants (H49Y, D614G, and T573I); these results supported by in silico studies should be further supported with experimental evidence.

## Results and discussion

**Figure 1:**
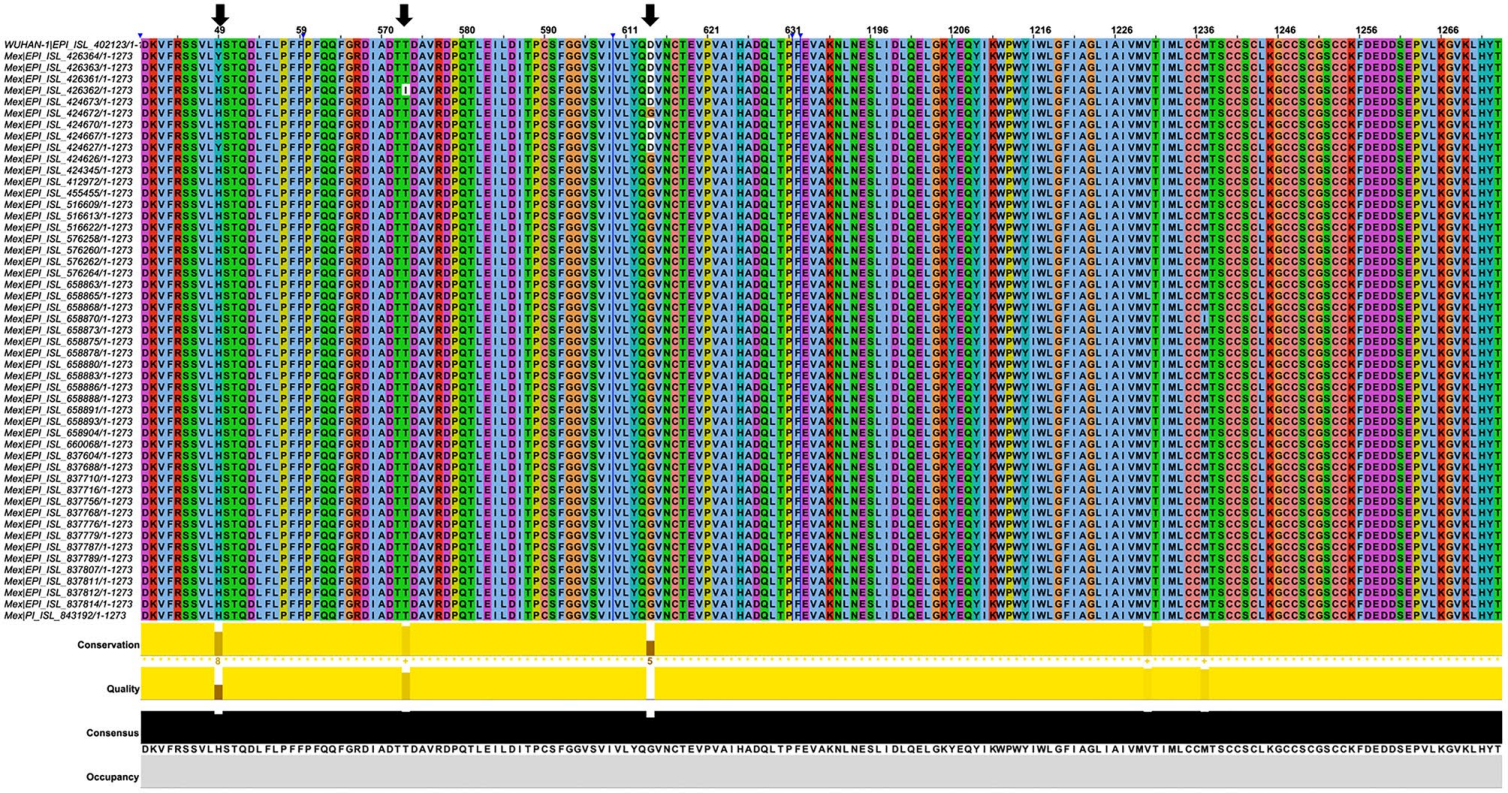
Multiple sequence alignment of Spike SARS-CoV-2 protein. The Wuhan Spike sequence was taking as wild type, the rest of the sequences correspond to Mexican Population. Arrows (black) indicate the row where the mutations were observed. The head of the arrows (blue) shows the hidden sequences.

Multiple sequence alignments of reported SARS-CoV-2 S proteins sequence show three mutants present in Mexican population (H49Y, T573I, and D614G) (Fig. 1, Table S1). These mutations mentioned above are far from the S protein’s RBD domain, a crucial domain for the virus interaction with the host cell. However, it is known that outside binding site mutations affect the ligand recognition as occurs with oseltamivir on neuraminidase from H1N1^34^.

D614G change is caused by an A/G nucleotide mutation at position 23,403 in the WT strain^35^, this mutant is being associated with higher viral loads and enhances viral infection effectivity in patients^8, 35, 36^. Despite the high viral loads, mutant G614 is neutralized by polyclonal antibody similarly to WT D614^35^. To date, this mutant has become the dominant circulating protein displacing the WT, according to the levels of mutations worldwide, presented on the nextstrain database (www.nextstrain.org). H49Y mutant is produced by C/T change at position 21,707 in the WT from S protein sequence^10^, producing a residue change from a positive histidine to an aromatic and polar tyrosine^9^ (H/Y). On the other hand, D614G mutant substitutions change from negative charge and high hindrance effects to none-radical group (H), which showed a stabilizing structure, suggesting a prevalent role in S protein evolution^13^. Finally, T573I mutant has no information available until now, but the change of T/I implies a modification from polar residue to non-polar hydrophobic residue changing the chemical environment to a more hydrophobic site. Even though these are punctual mutations due to the substitution’s chemical nature, perturbations at the structural and energetical level are expected.

### Molecular dynamics simulations

**Figure 2:**
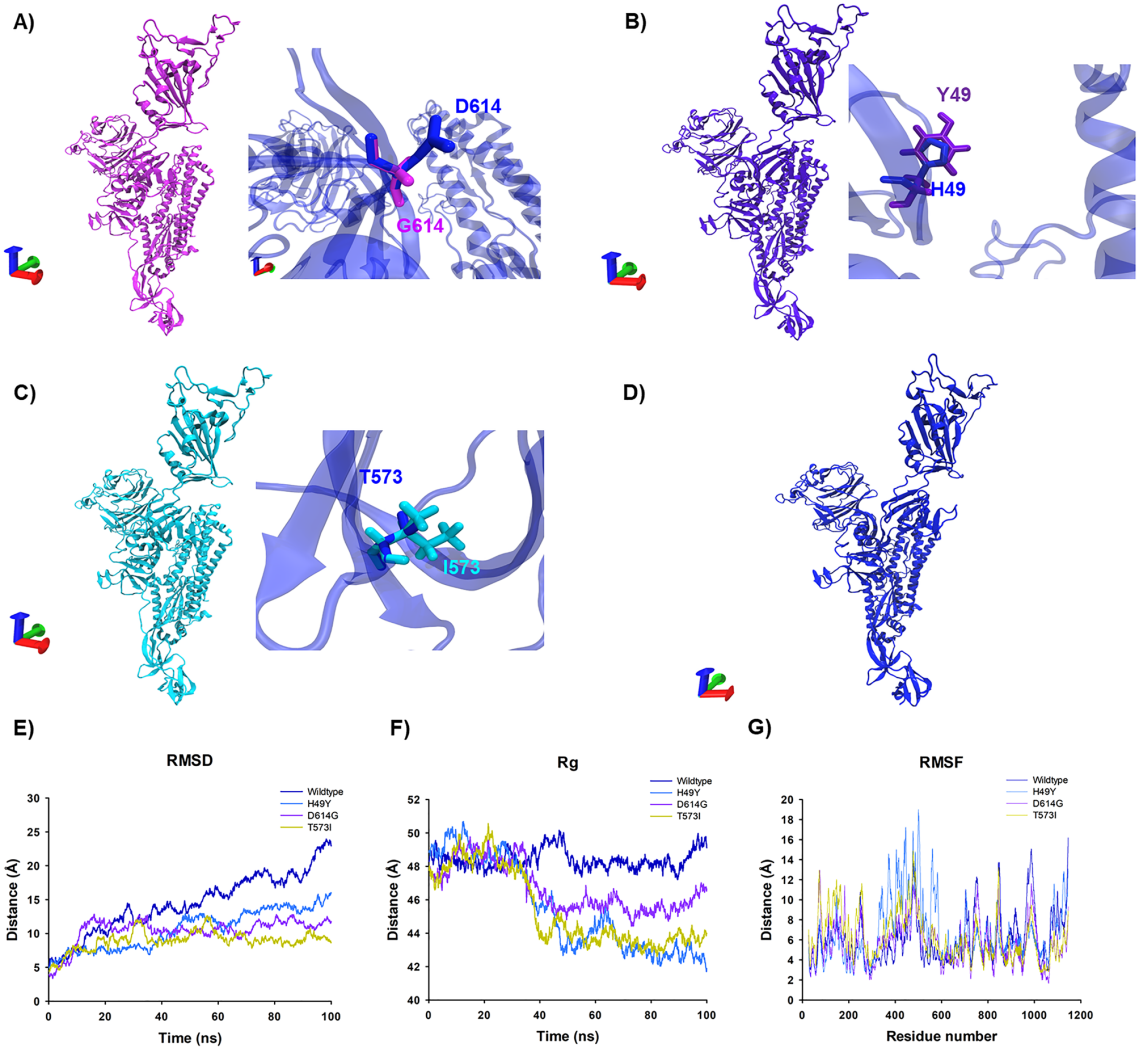
3D structure and trajectory from MD simulation of Spike protein of SARS-CoV-2 and variants. (**A**) 3D Structural conformation of D614G (pink) next, the zoom of the amino acid G614 is shown in pink while the WT residue is indicated in blue. (**B**) 3D structure of H49Y (purple), the zoom of the amino acid variant Y49 is shown in purple, the WT residue is shown in the sticks in blue. (**C**) 3D structure of T573I (cyan) the zoom of the residue variant I573 is shown cyan while the WT residue T573 is indicated in blue sticks. (**D**) 3D structure of wild type S protein is shown in blue. Axes: X, red; Y, green; Z, blue. The structural analysis was carried out by RMSD (**E**), Radius of gyration (**F**) and the RMSF (**G**). The trajectories of the WT are shown in navy blue, D614G are shown in purple, H49Y are shown in blue and T573I are shown in yellow.

The 3D structures of mutant S proteins were obtained by the punctual mutations of PDB: 6VSB crystal structure using PyMol software (Fig. 2); further they were submitted to MD simulations with Amber program. RMSD values were calculated to determine the average deviation in atomic stability under the MD simulations. All RMSD from the trajectories reached equilibrium after 40 ns of MD simulation, except the WT, which was oscillating during the rest of MD simulation (Fig. 2E). Our results are in agreement with other works where this behavior has been observed on the MD simulation of the Spike monomer^37^. The Rg values for WT showed expansion from 20 to 50 ns of MD with values from 47.5 to 50.02 Å; after this time, the Rg values are around 48 Å (± 1). D614G shows compactness from 35 to 50 ns with values from 46 to 45 Å, from 50 to 100 ns the Rg values are 45 Å (± 1), while T573I show compactness since the 25 to 40 ns of the trajectory with values from 47 to 43 Å, from 50 to 100 ns the Rg values are 43 Å (± 1), respectively. H49Y present compactness from 30 to 50 ns with values from 48 to 43 Å, in the last 30 ns remains around 45 Å (± 1) (Fig. 2F). Rg values suggest that variants have a similar degree of compactness while the WT shows an opposite behavior. We explored protein flexibility by RMSF values of the Cα from the MD simulations of the S proteins and its mutants (Fig. 2G). Eight main fluctuation peaks appeared as the most flexible areas of the WT protein, and these fluctuations were located between amino acids L226–T284, F400–D571, Q675–V722, K733–K776, K786–P812, G832–T866, A879–I1017 and V1065-D1146. In D614G mutant, the fluctuations were located between amino acids T63–D88, T95–Y200, P209–Y265, T323–P589, S735–K776, G832–L864, T961–T1017, and W1046–P1069. In H49Y, the most flexible residues were A67–D80, T108–S116, K129–Y170, V320–P589, I693–S721, D830–F855, and A1070–D1146. Finally, in T573I, the flexible regions were located at amino acids V62–Y204, P225–Y266, A363–N536, V736–A766, D830–L858, L962–T1009, and S1121–D1146 of the protein. The principal peak of all structure fluctuations is located on the Receptor Binding Domain (RBD) from F329 to P521. However, the highest values on this zone belong to H49Y and followed by T573I.

### Clustering analysis of apo-proteins

#### Wild type and mutants

**Figure 3:**
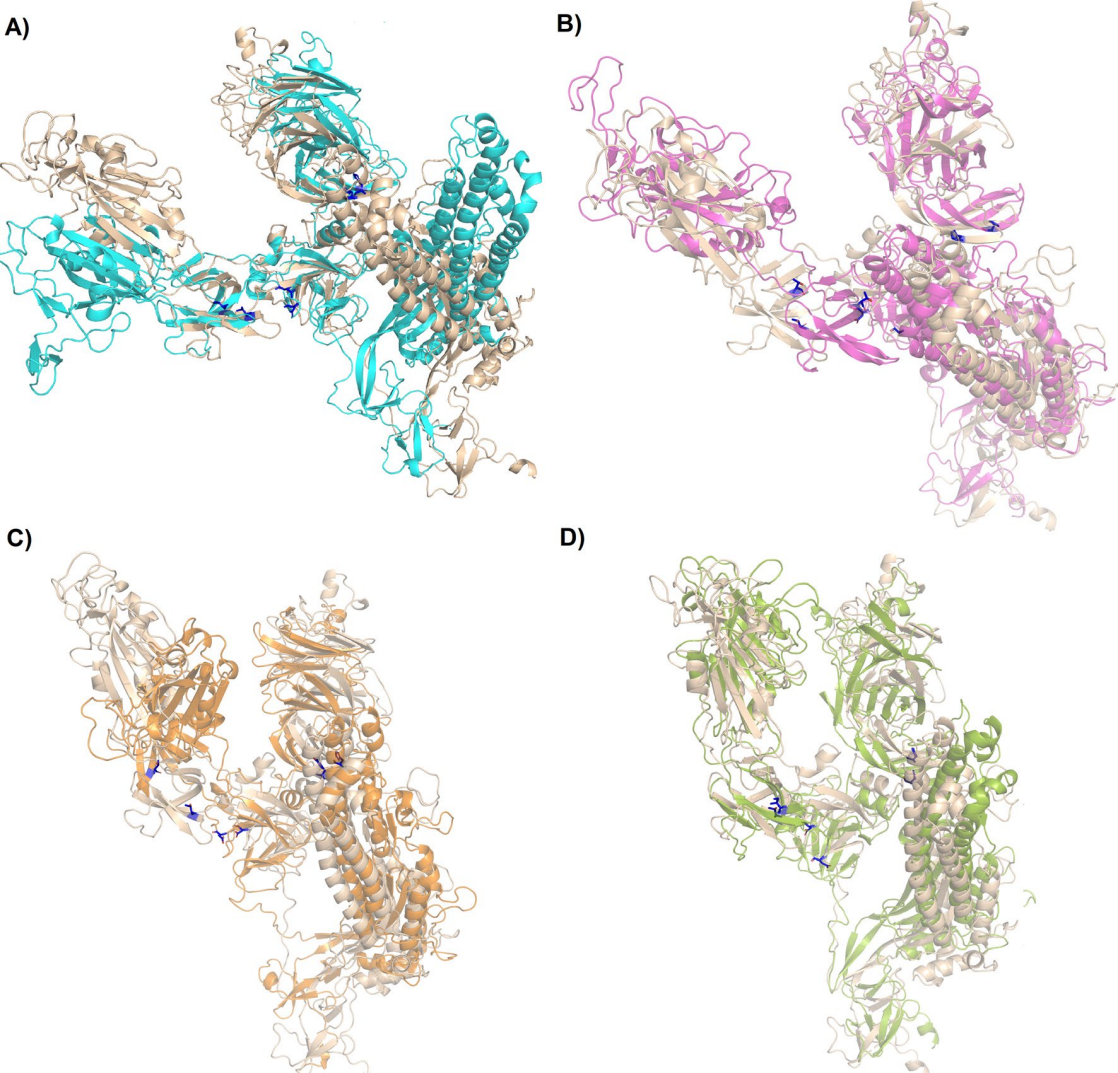
Structural alignment between the starting conformation and the most populated cluster on the trajectory of MDS. (**A**) WT, (**B**) D614G, (**C**) H49Y, and (**D**) T573I.

**Figure 4:**
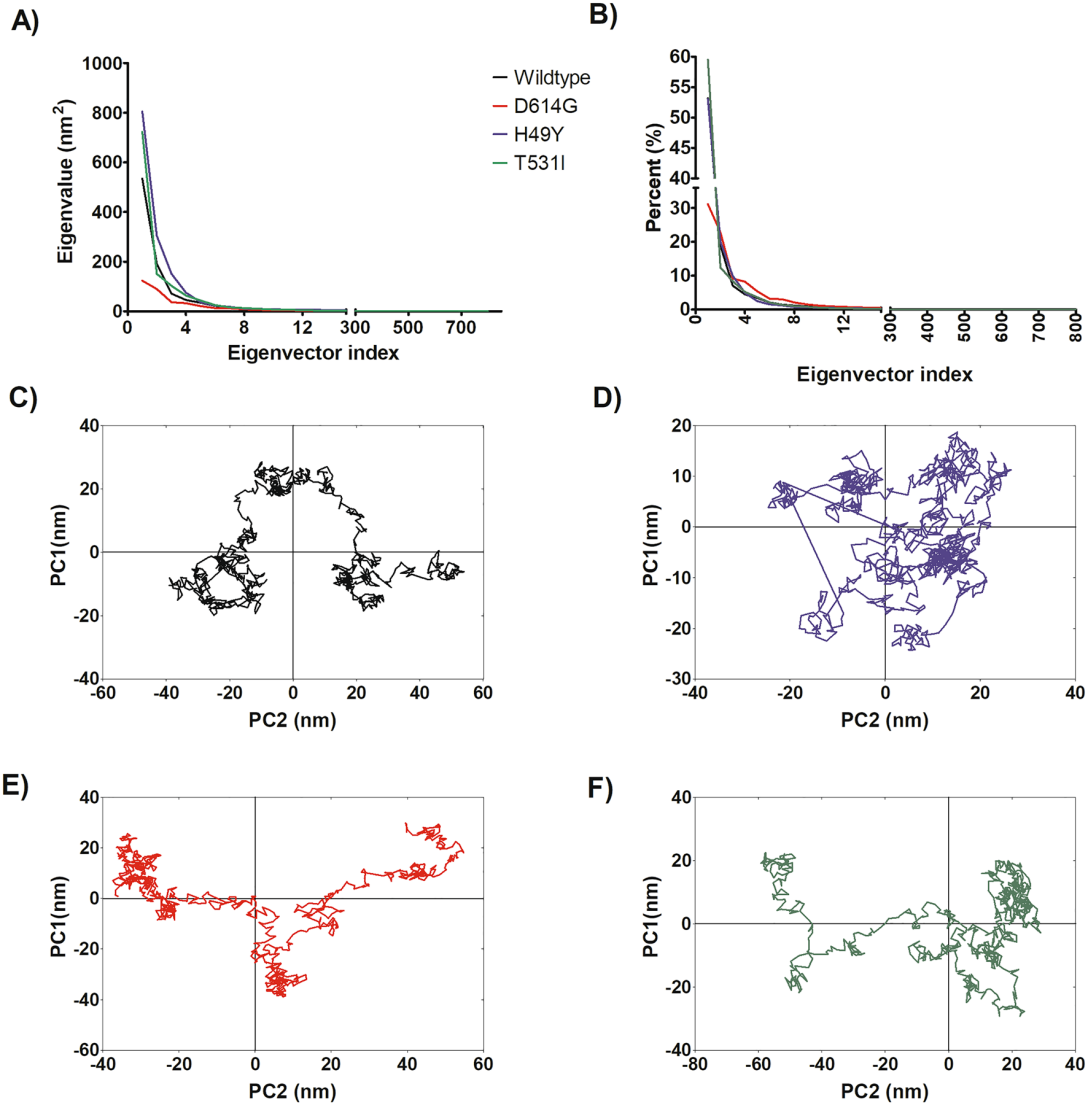
Principal component analysis of WT and mutated spike proteins. (**A**) eigenvectors of the covariance matrix, (**B**) percentage of each eigenvector vs eigenvalues, (**C**) projection of the motion in the phase space along the first and second eigenvectors (PC2 vs. PC1) of the WT, (**D**) projection of the motion in the phase space along the first and second eigenvectors (PC2 vs. PC1) of the D614G, (**E**) projection of the motion in the phase space along the first and second eigenvectors (PC2 vs. PC1) of the H49Y, (**F**) projection of the motion in the phase space along the first and second eigenvectors (PC2 vs. PC1) of T573I. Wild type spike protein is depicted in black, D614G in red, H49Y in green, and T573I in purple.

Representative ensembles were calculated from the last 80 ns of the MD simulation. 70% of the most populated conformations were grouped into the first 17, 15, 24, and 17 clusters for WT, D614G, H49Y, and T573I, respectively (Table S2); this clustering dispersion indicates that proteins possess a complex structural behavior. WT S protein showed similar clustering dispersion with D614G and T573I, while H49Y mutant showed higher clustering dispersion, which suggests it has complex structural behavior. Only the most populated cluster conformation was retrieved from each one of the systems, represented in Fig. 3. Regarding the WT structure, the most populated cluster conformation showed a higher difference regarding the initial conformation with an RMSD of 15.934 Å (Fig. 3A), followed by D614G (RMSD = 9.391 Å, Fig. 4B), H49Y (RMSD = 9.347 Å, Fig. 3C), and T573I (RMSD = 9.368 Å, Fig. 3D). Specifically, WT structural differences were observed in most of the structure, it is RBD region, HR1 (T912–L984) region, central helix (CH, D985–G1035) region, and small differences were observed from amino acids D287–F318 that are part of the N-terminal domain (NTD), and G550–T696 that include fusion peptide domain (FPD) belonging to the S1 subunit, in previous reports higher fluctuations on RBD region in monomeric and trimeric form were found^37, 38^; contrastingly, D614G mutant showed differences in regions spanning from D287–Q321 to T530–T696 which indicates that a single residue mutation affected the stability of the central portion of the S protein structure compared to the WT S protein. WT and D614G proteins exhibited differences in their secondary structure and displacements in loops, α-helices, and β-sheets structures, leading to a more compacted structure than WT S in agreement with our Rg findings. Contrastingly, the RBD region is more exposed, which is in line with previous observations where an increment between S1 and S2 distance is suggested to facilitate the viral attachment to the host cell, increasing its transmission mutant^39^.

While in the H49Y mutant, structural changes were observed in RBD and NTD domain compared to WT; H49Y induces fewer structural changes on the HR1 region, which correspond to what we observed in the above RMSD and RMSF. These results are consistent with the in silico predictions of energetic stability induced mainly by H49Y mutation and, to a lesser extent, by D614G mutant^13^. In T573I mutant, reduced fluctuations from amino acids I587–I720 were observed, which correspond to the central portion of the protein, this lead to a more compacted form of the protein, which is in line with the Rg observations; therefore, T573I mutation seems to induce structural stability compared to the WT protein as well as in the region around where the mutation is found.

### Principal component analysis

An exploration of the principal components that contribute to the global motions of WT S protein and mutant systems was performed (Fig. 4A) from the MD simulations. According to PCA analysis, the first 15 eigenvectors captured 91–97% of the total protein motions (95.43, 91.22, 96.88, and 96.59% for WT, D614G, H49Y, and T573I, respectively) (Fig. 4B). Whereas, the projections of the first two principal components (PC1 vs PC2) contributed to 54–72% of the collective motions (72.13, 54.02, 73.16, and 71.95% for WT, D614G, H49Y, and T573I, respectively) (Fig. 4B). By the projections of the essential subspace of PC1 vs. PC2, it was observed that the WT system (Fig. 4C) showed different mobility behavior compared to the mutant systems. Sampling different regions than the S protein mutants, which point out that the mutation of a single residue affects its interactions with their corresponding receptor and ligands^37^. On the other hand, D614G showed a more compact cluster distribution than the other systems, suggesting a reduction in conformational mobility due to single residue mutation; additionally, it showed a dissimilar conformer distribution along the subspace in comparison to the others systems, this behavior suggests that the trajectory sampled different regions of the phase space with different minima and small energy barrier, in this sense, D614G mutant affects the structural behavior of the protein, which is also reflected in the conformations observed during MD simulation (Fig. 4D). Similar cluster distribution was observed for H49Y and T573I mutants. H49Y showed slightly higher conformational mobility in comparison to T573I system (Fig. 4E,F).

**Figure 5:**
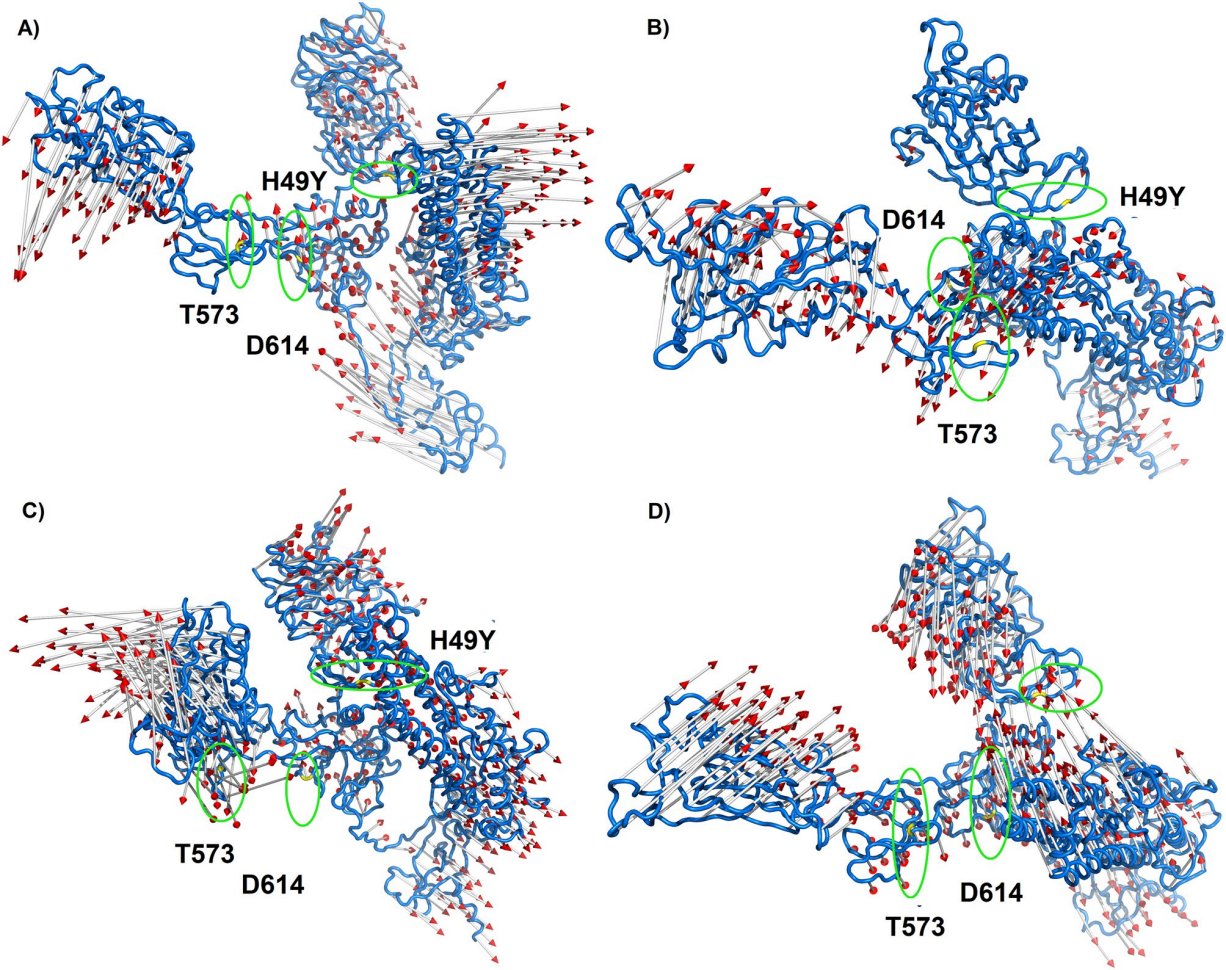
Graphical representation of the two extreme projections. Representation of the two extreme projections along the first eigenvector of MD simulation of (**A**) WT spike protein, (**B**) D614G, (**C**) H49Y and (**D**) T573I.

These results can be sustained by analyzing the graphical representation of the full mobility along PC1 and PC2 (Fig. 5), which allows us to study the direction and magnitude of the motions contributing to the total system's mobility. According to the projections, the motions of the WT system in the opposite direction, which provokes an expansion of the structure (Fig. 5A), which is in agreement with Rg results; i.e., RBD, NTD, HR1, CH and CD regions went in the opposite direction with higher magnitude. However, for D614G the direction of the movement changes in comparison to WT, the magnitude of the movement was higher for RBD region and S2 subunit, contrastingly for the NTD segment, a significantly minor detriment in the magnitude of the movements was observed (Fig. 5B), which corresponds to the cluster distribution of PC1 vs PC2 projection (Fig. 5D), this mutation not only increases the flexibility of the RBD region, but also changes its direction, which could contribute to an increase in virulence and could alter the binding affinity to the ACE receptor^13^. Previously, it was proposed that D614 forms a hydrogen bond with T859 and a salt bridge with K854 located in the S_2_ subunit of the other protomer; thus, the change of D by G could provide a flexible space between the monomers, which increases the protein's conformational flexibility as suggested by our MD simulations, this could lead to improved access to ACE2, and these events could explain the increase viral entry to the host cell^8, 40^. This motion perturbation is explained because D614 is located in an exposed loop at a side protein chain; it also has a negative charge, which will be a loss on the variant G614, which causes loss of interactions with other molecules or residues. The G614 has a neutral charge, is smaller, and introduces a more hydrophobic residue at this position of the protein, and this can result in loss of hydrogen bonds and/or disturb correct folding. Glycines are very flexible and can disturb the required rigidity of the protein at this position^13^. In the case of H49Y mutant, the whole mobility decreased, which was reflected in the magnitude of the porcupine representation in Fig. 5C. Moreover, the direction of the motions in the whole mutant protein was different in comparison to WT. Additionally, it was observed that the motions for NTD and S2 (816–1106) subunit were lesser in comparison to WT, suggesting that this mutant not only confers more structural stability as we have proven but also energetic stability as it was suggested by other authors^13^. Aside, it was found that the H49Y mutant virus has increased cell entry compared to WT S protein^8^.

Finally, the T573I mutation increases the magnitude of the protein’s movements at the S1 subunit; regarding the S2 subunit, it was observed that the direction of the movements is opposite to the WT (Fig. 5D). So, this mutation altered the magnitude and the direction of the protein’s mobility. For this mutant, there is not much more evidence about the virus behavior until now, so it should be explored to correlate with our findings.

### Molecular docking with known ligands

We performed molecular dockings with cepharanthine, hydroxychloroquine, and nelfinavir (known anti-SARS-CoV-2) on the experimental reported S protein to explore how these punctual mutations affect the protein–ligand binding inhibitors^20, 28, 32^.

**Figure 6:**
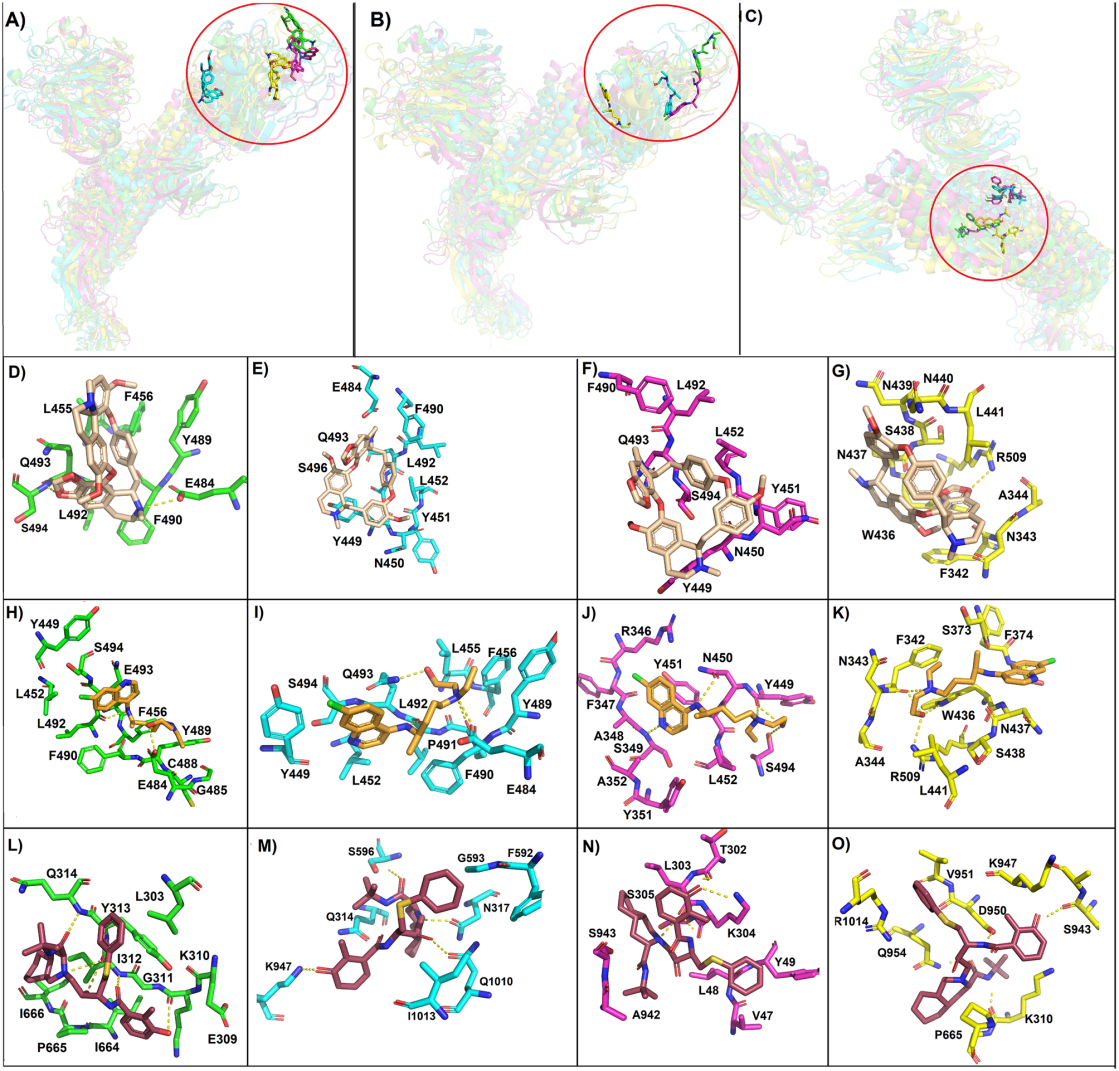
Molecular docking of most populated cluster conformation of spike proteins and mutants with experimentally proven compounds. General view of the binding pose obtained by docking of (**A**) cepharanthine, (**B**) hydroxychloroquine, (**C**) nelfinavir with wild type spike protein (green ribbon), D614G (cyan ribbon), H49Y (magenta ribbon) and T573I (yellow ribbon). Also, the interactions are between cepharanthine (wheat sticks) with (**D**) wild type spike protein, (**E**) D614G, (**F**) H49Y and (**G**) T573I; and hydroxychloroquine (orange sticks) with (**H**) wild type spike protein, (**I**) D614G, (**J**) H49Y and (**K**) T573I; and nelfinavir (raspberry sticks) with (**L**) wild type spike protein, (**M**) D614G, (**N**) H49Y and (**O**) T573I.

Cepharanthine showed potent antiviral activity against SARS-CoV-2 in vitro*,* and by molecular docking, S protein was suggested as its target. These experiments indicate that cepharanthine binds to RBD, interfering with the interaction of human ACE2 and the virus, avoiding the anchorage of the virus to the host cell^20, 41^. In WT complex, cepharanthine forms a hydrogen bond with E484 and S494 and hydrophobic interactions with L455, F456, Y489, F490, L492, and Q493 that are residues involved in the interactions between viral spike and ACE2 (Fig. 6A,D)^42^. Cepharanthine bound to WT spike protein with the highest binding free energy (− 6.57 kcal/mol), followed by H49Y mutant (6.42 kcal/mol) that also interacts with residues of the RBD (Y449, N450, Y451, L452, F490, L492, S494, and Q493) only by hydrophobic interactions (Fig. 6A,F). Cepharanthine from H49Y-cepharanthine complex was a little bit displaced compared to the WT-cepharanthine complex, which might be responsible for the binding energy reduction. While, D614G and T573I showed a decrease in binding free energy (− 5.95 and − 5.39, respectively). Also, hydrophobic interactions with Y449, N450, Y451, L452, E484, F490, L492, Q493 and S496 residues of the RBD domain of the D614G mutant were observed with cepharanthine (Fig. 6E). While within the T573I mutant, cepharanthine moved away from the typical interaction site and formed hydrogen bond with R509 and hydrophobic interactions with F342, N343, A344, W436, N437, S438, N439 and N440 none of these residues belong to ACE2-RBD interacting residues (Fig. 6G). It seems that T573I mutation might affect the binding affinity of this protein with cepharanthine and maybe the reason for the biological effect of cepharanthine as anti-SARS-CoV-2 agent^19^.

Hydroxychloroquine is a compound with effective in vitro inhibition activity against SARS-CoV-2 infection, it also elevates the endosomal pH and affects the ACE-2 terminal glycosylation avoiding the virus binding^25^. Two independent computational works suggested that hydroxychloroquine might interact with the RBD domain of spike protein, through it can disrupt the viral-host recognition and avoid viral infection^20, 28^. Therefore, in this study, we selected hydroxychloroquine to perform docking with WT spike protein and its mutants. Similar behavior as with cepharanthine was observed, hydroxychloroquine reaches its binding site on the RBD of the WT spike protein as well as on the D614G and H49Y mutants, while in T573I mutant hydroxychloroquine was moved away from this site, and it was reflected on the detriment of the binding free energy (Table S3, Fig. 6B). Hydroxychloroquine and WT S protein interact by forming hydrogen bonds between the compound and the amino acids Y489 and L492, and by hydrophobic interactions with Y449, L452, F456, E484, G485, C488, F490, E493 and S494 (Fig. 6H). The interaction of this compound with D614G is mediated by the formation of hydrogens bonds between the compound and amino acids E484, F490, L492 and Q493 and hydrophobic interactions with S494, Y449, L452, L455 and P491 (Fig. 6I). Interaction of the chemical compound hydroxychloroquine and H49Y mutant is mediated by the formation of hydrogen bonds with amino acids S349, Y449, N450, and S494 and by hydrophobic interactions with R346, F347, A348, Y351, A352, Y451 and L452 (Fig. 6J). In WT S protein and both D614 and H49Y mutants, hydroxychloroquine interacted with residues of the RBD that participate in ACE-RBD recognition^19, 42^. On the other hand, hydroxychloroquine and T573I mutant interacting residues were F342, W436, and R509, with which the compound formed hydrogen bonds and hydrophobic interactions were observed with amino acids S438, L441, N343, A344, S373, F374, N437, S438, which are not involved in ACE2–RBD interaction^42^ (Fig. 6K).

Nelfinavir is currently marketed as an anti-HIV drug, and recently, through molecular docking, it was shown to inhibit S protein-mediated fusion of SARS-CoV-2 with host cells^32, 41^. Nelfinavir interacts with S protein between the fusion peptide and HR1 helix near the S1/S2 cleavage site^32^. Therefore, nelfinavir was assayed by molecular docking against WT and mutants forms of the S protein. Nelfinavir was bound to WT S protein with the highest binding free energy (− 7.88 kcal/mol), followed by H49Y (− 7.52 kcal/mol), D614G (− 6.10 kcal/mol), and finally, T573I mutant (− 5.38 kcal/mol). Regarding the binding site, all ligands were allocated around HR1, FP region, and S1/S2 cleavage site, but nelfinavir reaches different binding sites in every S protein studied (Fig. 6C). Nelfinavir interacted with WT spike protein through the HR1 region and formed by hydrogens bond with amino acids, I312, and Q314, and hydrophobic interactions with amino acids L303, E309, G311, Y313, I664, P665 and I666 as previously reported (Fig. 6L)^32^. Furthermore, nelfinavir interaction with D614 mutant was also through the HR1 region, but the interacting amino acids were different than in the wild type docking, nelfinavir bound by the formation of hydrogen bonds with amino acids N317, S596, K947 and Q1010 and by hydrophobic interactions with amino acids Q314, F592, G593, I1013 (Fig. 6M). Whereas nelfinavir docking with H49Y mutant was through a couple of residues of the HR1 region and by hydrophobic interactions with amino acids (A942 and S943), in addition, nelfinavir also interacted with residues of the N-terminal domain of the S protein (S13–L303) and with amino acids (T302, L303, K304) by hydrogens bonds, and with residues (V47, L48, Y49, S305) by hydrophobic interactions, in this case, nelfinavir was displaced out from the HR domain more evidently than with the D614G mutant. Still, these interactions were energetically favorable (Table S3, Fig. 6N). In the case of nelfinavir-T573I mutant complex, interacted mainly with residues of the HR1 region by the formation of hydrogen bonds with amino acids S943, D950 and D954, and by hydrophobic interactions with K947, V951, R1014, it also formed a hydrogen bond with K310 and hydrophobic interaction with P665 (Fig. 6O), even though, this binding mode was energetically less favorable than those observed with other mutants and WT S protein.

Therefore, the ligand–protein interaction depicted a similar energy behavior than the observed with cepharanthine and hydroxychloroquine, where WT protein–ligand complex has the highest binding energy, while T573I-ligand complex has the lowest binding energy, which could indicate that the mutation on T573I position could be relevant for drug-spike protein interaction affecting not only the binding mode but also the binding free energy.

### MD simulation of the complex protein–ligand

To test the stability of the ligand-S protein complexes (WT, H49Y, D614G and T573I) obtained by molecular docking, 50 ns of MD simulation studies were carried out.

The average deviations in the atomic positions and stability through the trajectory of 50 ns of the MD simulations, the RMSD values of the protein–ligand complexes were calculated (Supplementary Information). The trajectories with cepharanthine show that the most stable trajectory at 50 ns corresponds to H49Y with values at 7 Å (± 1), while the trajectory of the WT is still unstable (Fig. S1A). The nelfinavir trajectories show the most stables trajectories with D614G; however, it was dissociated at the last five ns of the trajectory. The T573I complexes reach equilibrium at 25 ns with values at 9 Å (+ 1) (Fig. S1B). The WT complexes reach the equilibrium at 20 ns with values at 15 Å and remain without changes for the rest of the trajectory (Fig. S1B). From the trajectories of hydroxychloroquine with WT and the mutated proteins, it was observed that D614G complex form the most stable complex with values around 5 Å (± 1) (Fig. S1C), but this complex at the last 10 ns of the trajectory loses the equilibrium, whereas the trajectory with H49Y reach the equilibrium at 20 ns and remain for the rest of the trajectory with values at 8 Å (± 1) (Fig. S1C). The WT reaches the equilibrium at 20 ns with values at 10 Å (± 1) (Fig. S1C). The RMSF values of the trajectories with cepharanthine show that D614G exhibited the highest fluctuations on the RBD zone, follows by the WT, while T573I possesses the lowest fluctuations at this region (Fig. S1D). RMSF values with nelfinavir ligand present similar values for all the protein–ligand trajectories at the RBD, but shows an increase in fluctuation on the residues located at S1 for H49Y, WT, and D614G. In RMSF values for the trajectory with hydroxychloroquine, the T573I shows the higher values at RBD domain followed by the D614G, while the WT and H49Y have the lowest values.

### Binding free energy calculations of complex protein–ligand

Binding free energies (ΔG_mmgbsa_) of the protein–ligand complexes were calculated from the last 20 ns of the MD simulation once the system reached equilibrium (Table 1) using the MMGBSA approach. Regarding WT ligand complexes, Nelfinavir complexes were energetically more favorable than cepharanthine and hydroxychloroquine, which is in line with the predictions obtained by above molecular docking.

**Table 1:** Binding free energy components of complexes between ligands and SARS-CoV-2 spike protein (kcal/mol).

Complex	*ΔE*_*vdw*_	ΔE_ele_	ΔG_ele,sol_	ΔG_npol,sol_	ΔG_mmgbsa_
**Cepharanthine**					
D614G	− 32.15 (4.5)	− 11.43 (1.9)	31.18 (8.0)	− 3.96 (0.50)	− 16.36 (3.7)
H49Y	− 32.15 (3.8)	− 1.9 (0.2)	18.29 (6.0)	− 3.74 (0.30)	− 19.43 (3.3)
T573I	− 32.26 (5.9)	− 14.41 (0.4)	31.68 (7.0)	− 3.83 (0.70)	− 18.83 (5.1)
WT	− 24.90 (4.0)	− 51.19 (11.0)	63.53 (10.0)	− 2.97 (0.55)	− 15.53 (3.2)
**Hydroxychloroquine**					
D614G	ND	ND	ND	ND	ND
H49Y	− 18.43 (4.0)	− 15.05 (5.0)	21.93 (5.0)	− 2.49 (0.40)	− 14.05 (3.7)
T573I	− 20.50 (4.0)	− 11.18 (1.0)	18.87 (7.0)	− 2.71 (0.75)	− 15.53 (4.0)
WT	ND	ND	ND	ND	ND
**Nelfinavir**					
D614G					> 0
H49Y	− 23.42 (4.0)	− 66.49 (10.0)	79.13 (10.0)	− 3.18 (0.50)	− 13.96 (3.6)
T573I	− 44.55 (3.0)	− 116.60 (12.0)	127.29 (10.0)	− 5.47 (0.40)	− 39.34 (3.6)
WT	− 27.12 (3.6)	− 61.49 (9.0)	72.38 (8.0)	− 3.29 (0.55)	− 19.52 (3.3)

All energies are averaged over 200 snapshots at time intervals of 100 ps from the last 20 ns-long MD simulations, and they are in kcal/mol (± standard deviation). ND due to ligand diffuses of the binding pose at the first ns of simulations.

Comparing among mutants, it can be seen that nelfinavir-T573I complex was energetically more favorable than the corresponding with WT-nelfinavir complex (− 39.34 vs − 19.52 kcal/mol), followed by nelfinavir-H49Y complex, in whose case the binding free energy becomes more positive, indicating a lesser favored complex (− 13.96 vs − 19.52 kcal/mol). While the nelfinavir-D614G complex was not energetically favorable (Table 1).

In the case of cepharanthine complexes, the WT-cepharanthine complex (− 15.53 kcal/mol) was the least favorable in comparison to the corresponding complex with mutant proteins, where the most favorable complex was formed with H49Y (− 19.43 kcal/mol), followed by T573I (− 18.83 kcal/mol) and D614G (− 16.36 kcal/mol).

For Hydroxychloroquine complexes, those formed with WT and D614G were diffused, indicating that a favorable complex was not formed. Only energetically favorable complexes were formed with T573I (− 15.53 kcal/mol) and H49Y (− 14.05 kcal/mol), in which case is found among the least favorable complexes, this points out that hydroxychloroquine did not form stable complexes with WT spike protein and D614G- Pandey et al*.,* found by molecular docking that hydroxychloroquine has lower affinity by WT S protein, which could be due to the labile and bulkier carbon chain, which disturb hydroxychloroquine binding reducing in this way the binding site^29^. On the other hand, Fantini et al*.,* found by MD simulation studies that hydroxychloroquine rather than interact with Spike protein, it is bound to gangliosides site near to ACE-2, saturating the spike protein binding sites^43^. However, it is interesting how T573I and H49Y mutant produce more energetically favorable complex, so further experimental studies regarding these other spike inhibitors deserve to be experimentally studied.

More detailed analysis showed that non-polar interactions (ΔE_nonpolar_ = ΔE_vwd_ + ΔG_npol,sol_) mainly contributed to the free energy of binding, where van der Waals interactions were the component with the most important contribution in comparison to non-polar solvation term (ΔG_npol,sol_). Contrastingly, the contribution of polar solvation term was stronger unfavorable that screen the small favorable contribution of the electrostatic term (*ΔE*_*ele*_). These findings suggest that the studied compounds mainly interacted by hydrophobic interactions.

### Non-covalent interaction analysis of the protein–ligand complexes

**Figure 7:**
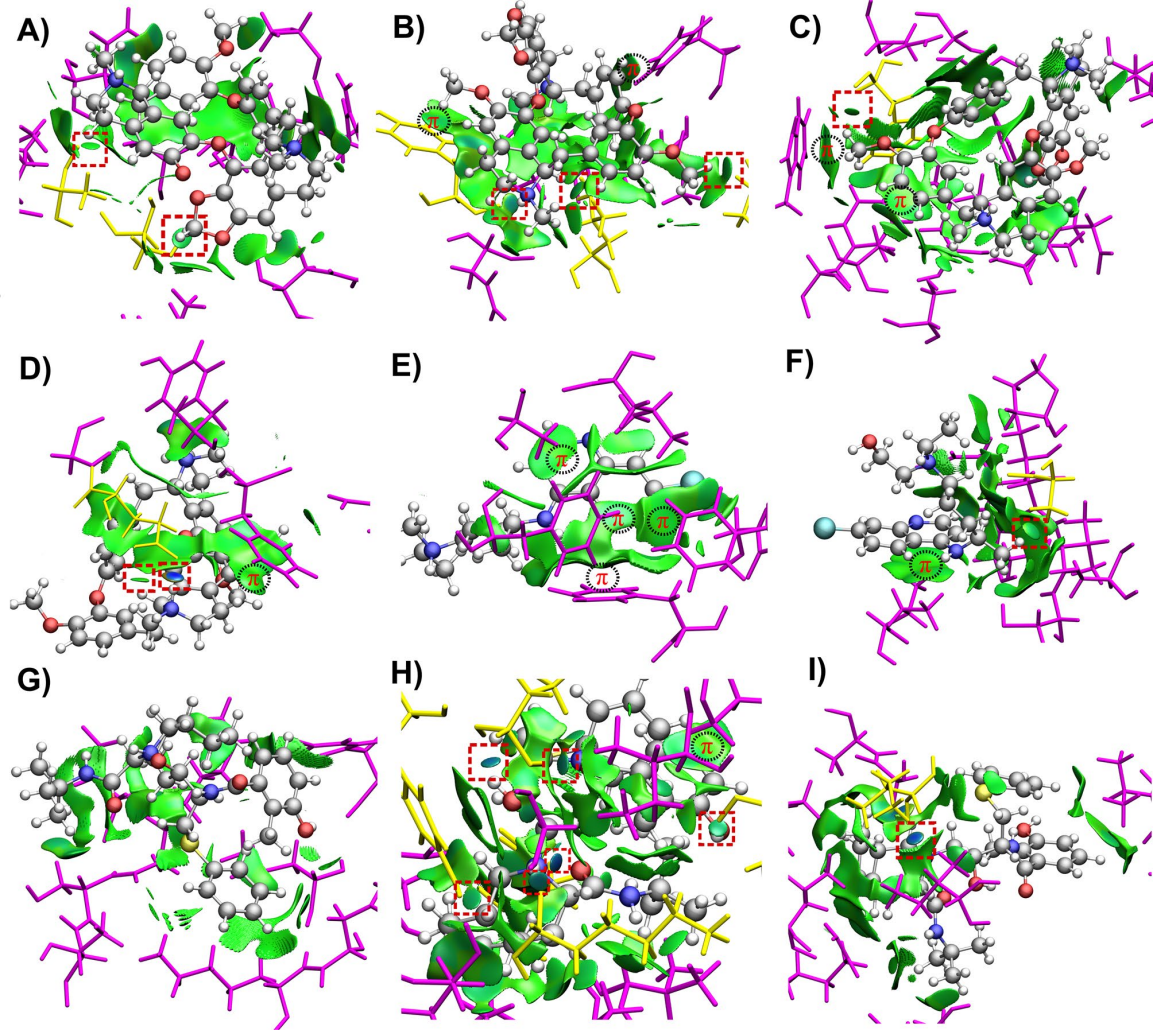
The NCI isosurfaces (drawn at 0.5) of (**A**) Cepharanthine-D614G, (**B**) Cepharanthine-H49Y, (**C**) Cepharanthine-T573I, (**D**) Cepharanthine-WT, (**E**) Hydroxychloroquine-H49Y, (**F**) Hydroxychloroquine-T573I, (**G**) Nelfinavir-H49Y, (**H**) Nelfinavir-T573I and (**I**) Nelfinavir-WT complexes. Blue and green regions represent strong attractive and weak dispersive interactions, respectively. The localized interactions are marked in a red square. The regions of π-interactions are also indicated. The amino-acid residues forming localized and delocalized interactions with the ligand are depicted in yellow and magenta, respectively.

Most populated cluster conformations of each of the protein–ligand complexes were obtained through clustering analysis to gain the map of interactions established between the compounds and S protein WT and mutants after being submitted to MD simulation. To this end, the Non-Covalent Interaction index (NCI)^44^ was employed. NCI has become a useful tool for the qualitative and quantitative analysis of intermolecular interactions in supramolecular and biological systems^45–47^. The NCI framework belongs to quantum chemical topology^48^, and has the advantage of going far beyond the simple determination of non-covalent interactions by geometrical examination. NCI provides an intuitive way to visualize the presence of non-covalent interactions by the analysis of 3D isosurfaces^49^, such as those observed in Fig. 7. Localized interactions, i.e., those that can be attributed to some atomic pairs (e.g., hydrogen bonds), are depicted as small disk surfaces that appear in a middle way between the interacting atoms. On the other hand, delocalized interactions (for example, van der Waals forces) emerge as flat extended surfaces that cover large interacting regions. Additionally, a color scale is used to characterize the interaction strength. Bluish, greenish and reddish zones of the NCI isosurfaces represent strong attractive, weak and repulsive interactions, respectively. Furthermore, a quantitative analysis is obtained by the integration of the electron density in the NCI isosurfaces and its partition in different ranges of interactions (strong attractive, weak and repulsive). These quantities have been shown to correlate with binding energies in protein–ligand interactions^50^. More details about NCI theory and interpretation can be found in the Supplementary Information.

The NCI surfaces of the most populated cluster conformations of the protein–ligand complexes are depicted in Figs. 7 and S2. The classification of the interactions between the ligands and the closest residues of the S protein, according to NCI, is given in Table NCI. In addition, the atom–atom intermolecular distances of the localized interactions are shown as a complementary guide for its strength classification. The residues forming localized and delocalized interactions with the ligand are depicted in yellow and magenta, respectively. The surfaces corresponding to localized interactions are marked by a red square. The regions where π-interactions are present are indicated as well. As a general trend, it is observed that the protein–ligand complexes are stabilized mainly by dispersive interactions, i.e., the green surfaces predominate. This result is supported by the NCI integrals, which show that the contributions from weak interactions are larger than those of the strong attractive ones (Fig. S2). This outcome is also in agreement with the previous conclusion drawn from the above MMGBSA approach, that non-polar interactions have a major contribution to the free energy of binding. Moreover, it is interesting to note that the total NCI integrals show, in the main, the same tendency that the free energies of binding (Fig. S2). Nevertheless, despite the fact that localized interactions are less abundant, because of their directional nature, they possess an important role in determining the orientation of the ligand in the protein^51^.

Cepharanthine remains bound to the RBD in WT via a strong N–H⋯O hydrogen bond and a weak non-conventional C–H⋯O hydrogen bond^52^ to E484. Besides, one of the methyl groups of cepharanthine is forming a C–H⋯π interaction with the aromatic ring of F490. As well, it forms van der Waals contacts with four close neighbors (Table 2). A diversity of other interactions is found for this ligand with the other mutant-S proteins. With D614G, it is slightly displaced with respect to the initial site (Fig. S3). Two new local interactions, the so-called hydrogen–hydrogen bonds (H⋯H)^53^ are formed instead, while delocalized interactions are established with eight neighbors. In its complex with H49Y, cepharanthine is still bound to the same place as at the beginning of the MD simulation. There were found a strong N–H⋯O hydrogen bond with Y449 and two H⋯H bonds with L542 and T470, respectively. In addition, another C–H⋯π interaction is formed between a methyl group of the ligand and the aromatic ring of Y449. It also forms a C–H⋯π interaction with the same residue than in WT, but the ligand acts as an acceptor in this case. The number of van der Waals contacts is also similar as in its complex with WT (Table 2). Finally, in the Cepharanthine-T573I complex, cepharanthine was slightly moved away from the initial binding site. A weak C–H⋯O hydrogen bond with F342 is formed. Additionally, two C–H⋯π interactions are established between a methyl group of the ligand and W436, and between an aromatic group of the ligand and F374, respectively. For this complex, nine van der Waals interactions were found.

**Table 2:** The localized and delocalized interactions found for each protein–ligand complex within the NCI isosurfaces.

System	Localized interaction/residue (strength/distance)	Delocalized interaction/residue
**Cepharanthine**		
D614G	H⋯H/S349 (weak/2.94 Å); H⋯H/A352 (weak/2.28 Å)	Van der Waals/A344; Van der Waals/N354; Van der Waals/R346; Van der Waals/N450; Van der Waals/F347; Van der Waals/L452; Van der Waals/A348; Van der Waals/I468
H49Y	N–H⋯O/Y449 (strong/2.05 Å); H⋯H/L452 (weak/2.13 Å); H⋯H/T470 (weak/2.26 Å)	C–H⋯π/Y449; Van der Waals/L492; Van der Waals/N450; Van der Waals/Q493; Van der Waals/L452; Van der Waals/S494; C–H⋯π/F490
T573I	C–H⋯O/F342 (weak/2.91 Å)	Van der Waals/L335; Van der Waals/L368; Van der Waals/F338; Van der Waals/S371; Van der Waals/G339; Van der Waals/S373; Van der Waals/N343; C–H⋯π/F374; Van der Waals/D364; C–H⋯π/W436; Van der Waals/V367
WT	N–H⋯O/E484 (very strong 1.71 Å); C–H⋯O/E484 (weak/3.05 Å)	Van der Waals/E484; C–H⋯π/F490; Van der Waals/G485; Van der Waals/Q493; Van der Waals/Y489
**Hydroxychloroquine**		
H49Y		π⋯π (parallel displaced)/F338; Van der Waals/G339; C-H⋯π/A372; C–H⋯π/F342; C-H⋯π/F374; Van der Waals/L368
T573I	H⋯H/A522 (weak/2.11 Å)	C–H⋯π/I332; Van der Waals/A522; Van der Waals/N360; Van der Waals/T523; Van der Waals/C361; Van der Waals/V524; Van der Waals/C391; Van der Waals/C525; Van der Waals/P521
**Nelfinavir**		
H49Y		Van der Waals/F817; Van der Waals/D936; Van der Waals/L821; Van der Waals/S939; Van der Waals/N824; Van der Waals/S940; Van der Waals/K825; Van der Waals/T941, Van der Waals/Q935
T573I	N–H⋯O/K310 (very strong/2.05 Å); O–H⋯O/I312 (very strong/1.96 Å); N–H⋯O/I312 (strong/2.14 Å); C–H⋯O/Y313 (weak/2.33 Å); O–H⋯O/D663 (strong/2.22 Å); N–H⋯O/Q954 (very strong/1.88 Å)	Van der Waals/E309; Van der Waals/D950; Van der Waals/G311; Van der Waals/V951; Van der Waals/Y313; Van der Waals/N953; Van der Waals/I664; Van der Waals/Q957; C–H⋯π/P665
WT	N–H⋯O/I666 (very strong/1.87 Å)	Van der Waals/I312; Van der Waals/G667; Van der Waals/Q314; Van der Waals/A668; Van der Waals/L611; Van der Waals/V1040; Van der Waals/Q613; Van der Waals/D1041; Van der Waals/A647

The hydroxychloroquine complexes were only stable with the H49Y and T573I mutants, where they migrate from different pockets of the RBD regions. In the case of WT and the D614G mutant, the ligand diffuses from the proteins. The NCI isosurfaces reveal that for these systems, localized interactions are scarce. This fact explains why these complexes show the lowest free energy of bindings according to the MMGBSA approach. The π-interactions govern the complexation of hydroxychloroquine with H49Y. It forms a distant π⋯π stacking with the aromatic ring of F338, while the two aromatic rings of the ligand act as acceptors in C–H⋯π interactions formed with the A372 and F374 residues. In addition, one of the C–H bonds from the aromatic ring of the ligand act as a donor in another C–H⋯π contact established with the aromatic ring of F342. Two more van der Waals contacts were found for this system (Table 2). The hydroxychloroquine-T573I complex shows one H⋯H weak hydrogen bond with A522 and act as an acceptor for a C–H⋯π interaction formed with I332. With the remaining close residues only van der Waals forces are established (Table 2).

The nelfinavir complex with WT shows large free energy of binding, which can be partially attributed to a strong N–H⋯O hydrogen bond formed with I666. It further has van der Waals interactions with other nine residues, which contribute to form a stable complex since the ligand did not move from the original binding site. Contrarily, its complex with D614G was not energetically favorable (Table 1). Interestingly, the Nelfinavir-H49Y complex also shows nine van der Waals contacts but no localized interaction. This outcome explains why it shows one of the lowest free energies of binding among all the analyzed complexes. The residues that interact with nelfinavir were localized in the HR1 region. The nelfinavir-T573I has a greater number of localized interactions. It forms one strong N–H⋯O hydrogen bond with K310, one N–H⋯O and one O–H⋯O hydrogen bond with I312, another N–H⋯O hydrogen bond with Q954, and a strong O–H⋯O hydrogen bond with D663, plus a weak C–H⋯O hydrogen bond with Y313. Also, it acts as an acceptor in a C–H⋯π interaction established with P665. Finally, van der Waals forces are also abundant for this complex, which provides extra stabilization. This analysis explains why the Nelfinavir-T573I system has such great free energy of binding compared to the rest of the complexes, which is also predicted by the NCI integrals.

## Materials and methods

### Multiple alignments of spike (S) protein

The sequences corresponding to S protein from SARS-CoV-2 were downloaded (April 27th) from the Global Initiative for Sharing All Influenza Data (GISAID) database^54^ (www.gisaid.org). The sequence from Wuhan was used as WT, and it was aligned against 49 S sequences found in Mexican population (Table S1). Multiple alignments were carried out in ClustalX using default parameters, further, it was edited on Jalview^55^. The whole sequence of the S protein has 1282 residues, we showed only the residues where the protein exhibited mutations found in the Mexican population, the sequences without changes were hidden. To obtained the 3D mutants of S protein, mutations were done on PyMol^56^, using as a template the crystal structure of SARS-CoV-2 S glycoprotein (PDB: 6VSB). Visualization of wild type and the H49Y, T573I, and D614G S mutants were performed on VMD (Fig. 2). Zoom of the structural 3D alignments were done to visualize the region which contains the mutations (Fig. 2).

### MD simulation and analysis

MD simulations were carried out with AMBER 16 software package^57^ using ff14SB forcefield^58^. Force field ligand parameters were assigned using the semi-empirical AM1-BCC and the general Amber force field (GAFF)^59^. The systems were solvated using an explicit TIP3P water model and centered into a rectangular box of 12.0 Å; after that, all the systems were neutralized by adding 6 Na^+^ counter ions. Each one of the systems was minimized through 2500 steps of steepest descent and 2500 steps of conjugate gradients. Then, they were equilibrated through 500 picoseconds (ps) of heating and 500 ps of density equilibration with weak restraints followed by 2 ns (ns) of constant pressure equilibration at 310 K. MD simulations of Apo proteins were carried out for 100 ns while the complex ligand–protein complexes MD simulations of Apo proteins were carried out for 100 ns while the complex ligand–protein complexes for 50 ns, under periodic boundary conditions and using an NPT ensemble at 310 K. The electrostatic term was described through the particle mesh Ewald method^60^; using a 10.0 Å cut-off, similar radio was chosen for van der Waals interactions. The SHAKE algorithm^61^ was used to constrain bond lengths at their equilibrium values, and a time step was set to 2.0 fs. Temperature and pressure were maintained with the weak coupling algorithm^62^ using coupling constants τT and τp of 1.0 and 0.2 ps, respectively (310 K, 1 atm). Trajectories were analyzed using cpptraj module for root mean squared deviation (RMSD), root mean squared fluctuations (RMSF), the radius of gyration (R_g_).

Based on the knowledge of the equilibration time, clustering analysis (using a 3.0 Å cut-off) was done using a hierarchical agglomerative (bottom-up) approach employing AmberTools 16, where the most populated cluster conformation of each MD simulation was obtained, and further structural examination was performed, in order to compare the structural differences between the initial conformation and the one obtained after MD simulation. Clustering analysis is a statistical method data mining tool that allows partitioning a data set based on similar features; in MD simulations it allows to reduce complex ensembles getting smaller subsets of data and obtain representative conformation from individual clusters^63, 64^, in this case, we pick up a conformation from the most populated cluster Principal component analysis (PCA), also known as essential dynamic (ED) was performed. PCA analysis is a statistical technique that allows obtaining the large scale collective motions of the atoms on the simulations, which are frequently correlated to the biological function and biophysical properties^65^. The performed method is described in detail elsewhere^66^. Structural analysis of the systems and figures were performed using PyMOL v0.99^56^.

Molecular graphics were performed in SigmaPlot 12.0, and protein visualization was performed by VMD and Chimera^67, 68^.

### Binding free energies calculation

The MMGBSA^69–71^ approach was employed to calculate the binding free energy (ΔG_bind_). Twenty hundred snapshots at time intervals of 100 ps were selected through the equilibrated simulation (last 20 ns), and all counterions and water molecules with a salt concentration of 0.10 M were deleted using the implicit solvent model^72^. ΔG_bind_ analysis was performed as previously described^73^.

### Molecular docking

Autodock 4.2 software was used for docking calculations^74^. Focused docking was performed, for cepharanthine and hydroxychloroquine grid box was center on RBD domain with a grid box size of 80 Å × 70 Å × 70 Å, as reported elsewhere^20, 28^. For nelfinavir, grid box was focused on residues L303, Y313, Q314, between HR1 and FP region. With a grid box size of 80 Å × 82 Å × 80 Å^32^ and a grid spacing of 0.375 Å. Compounds and S proteins were prepared using AutoDock Tools 1.5.6^74^. Polar hydrogen atoms and Kollman charges were encompassed for the S protein. Lamarckian genetic algorithm was used as a scoring sample with a randomized population of 100 individuals, and energy evaluations of 1 × 10^7^, 100 runs were performed. As a selection criterion, the conformation with the lowest free energy values was chosen. Docking results were analyzed using Autodock Tools 1.5.6^74^, and whereas figures were further processed with Pymol v.099^56^. Ligand structures are provided in Supplementary Material as in Fig. S4.

### Non-covalent interaction index

The NCI isosurfaces were generated using promolecular electron densities, ρ(r), taking as input the geometry obtained from the most populated cluster conformation retrieved from MD simulations. The keyword LIGAND was used to analyze exclusively the intermolecular interactions between the drugs and the surrounding amino acid residues, where a 5 Å radio cut-off was applied. The integrals of ρ(r) in the NCI isosurfaces were split in different ranges of sign(λ)ρ(r), where sign(λ) is the sign of the second eigenvalue of the electron density Hessian. These are: from − 0.1 to − 0.02 for strong attractive regions, from − 0.02 to 0.02 for van der Waals regions, and from 0.02 to 0.01 for repulsive regions (all values in atomic units [a. u.]). The results from the last interval were omitted because their values were negligible. All the calculations were performed with the NCIPLOT4 software^75^, and the figures were created with VMD 1.9.3^67^.

## Supplementary Information

Supplementary Information.
