# Supplementary material for: Structural insights into SARS-CoV-2 spike protein and its natural mutants found in Mexican population
Source: Sci Rep. 2021 Feb 25;11:4659. doi: 10.1038/s41598-021-84053-8 (PMC7907372; doi:10.1038/s41598-021-84053-8)
Supplement: Supplementary file 1 — Supplementary Information. [file 41598_2021_84053_MOESM1_ESM.docx]

***Supplementary Information***

Structural insights into SARS-CoV-2 spike protein and its natural mutants found in Mexican population.

**Authors:** Yudibeth Sixto-López^1^, José Correa-Basurto^1^, Martiniano Bello^1^, Bruno Landeros-Rivera^2^, Jose Antonio Garzón-Tiznado^3^ and Sarita Montaño^3^

**Affiliation:**

^1^ Laboratorio de Diseño y Desarrollo de Nuevos Fármacos e Innovación Biotecnológica (Laboratory for the Design and Development of New Drugs and Biotechnological Innovation), Sección de Estudios de Posgrado e Investigación, Escuela Superior de Medicina, Instituto Politécnico Nacional, Plan de San Luis y Salvador Díaz Mirón s/n, Casco de Santo Tomás, Ciudad de México 11340, México

^2^ CNRS, Laboratoire de Chimie Théorique, LCT, Sorbonne Université, Paris, France

^3^ Laboratorio de Bioinformática y simulación molecular, Facultad de Ciencias Químico Biológicas, Universidad Autónoma de Sinaloa, Culiacán Sinaloa México.

**Corresponding author:** Sarita Montaño email: [mmontano@uas.edu.mx](mailto:mmontano@uas.edu.mx)

| **Contents** | **Page No.** |
| --- | --- |
| Figures | **3** |
| Tables | **7** |
| Non-Covalent Interaction index (NCI) | **12** |

**TABLE OF CONTENTS**

**Figures**


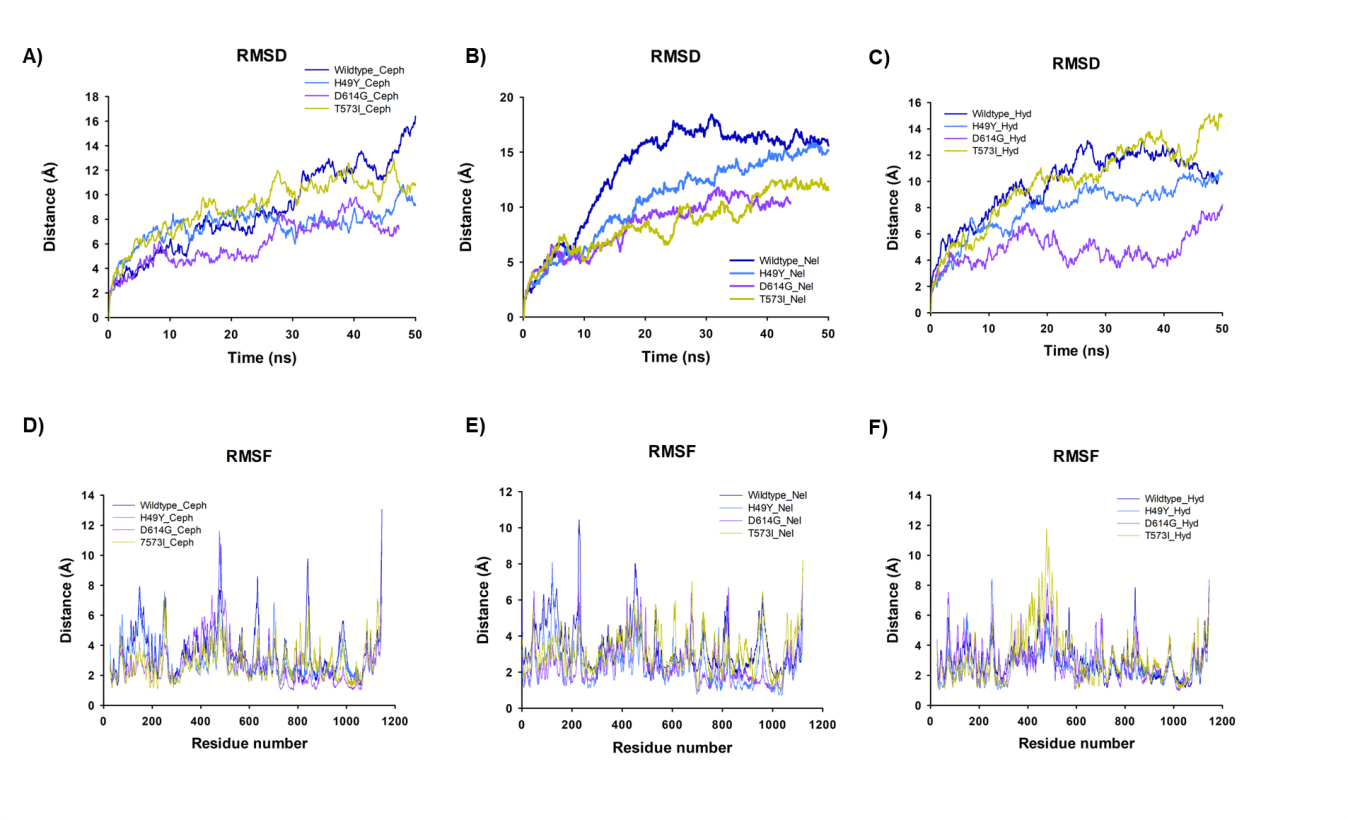


**Figure S1. The trajectory of holo proteins**. A) The RMSD with cepharanthine. B) RMSD with nelfinavir. C) RMSD with hydroxychloroquine. D) RMSF with cepharanthine. E) RMSF with nelfinavir. F) RMSF whit hydroxychloroquine.


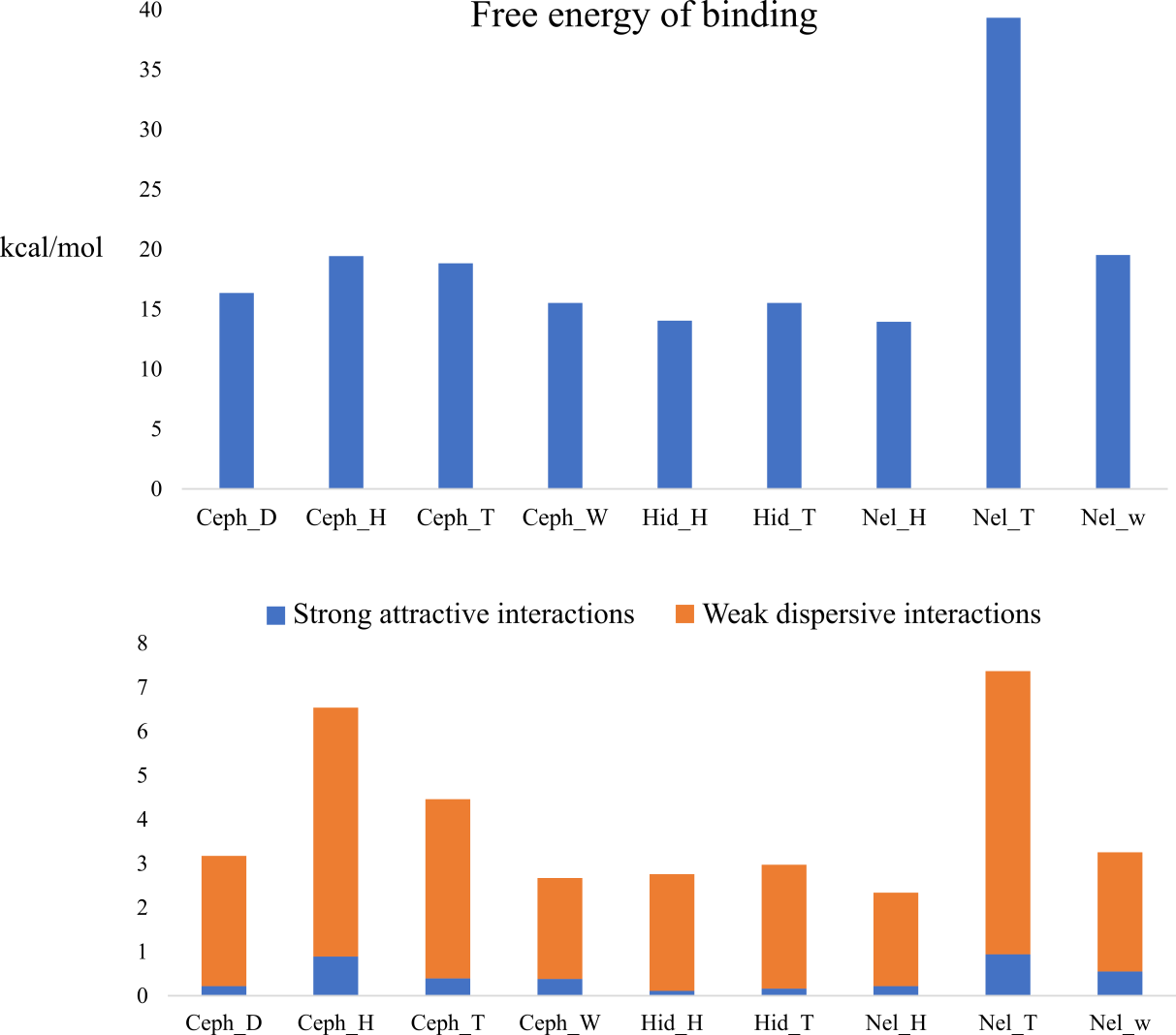


**Figure S2.** Free energy of binding (top) and NCI integrals (bottom) for the 9 protein-ligand complexes studied in this work. The NCI integrals are decomposed in strong attractive and weak dispersive interactions.


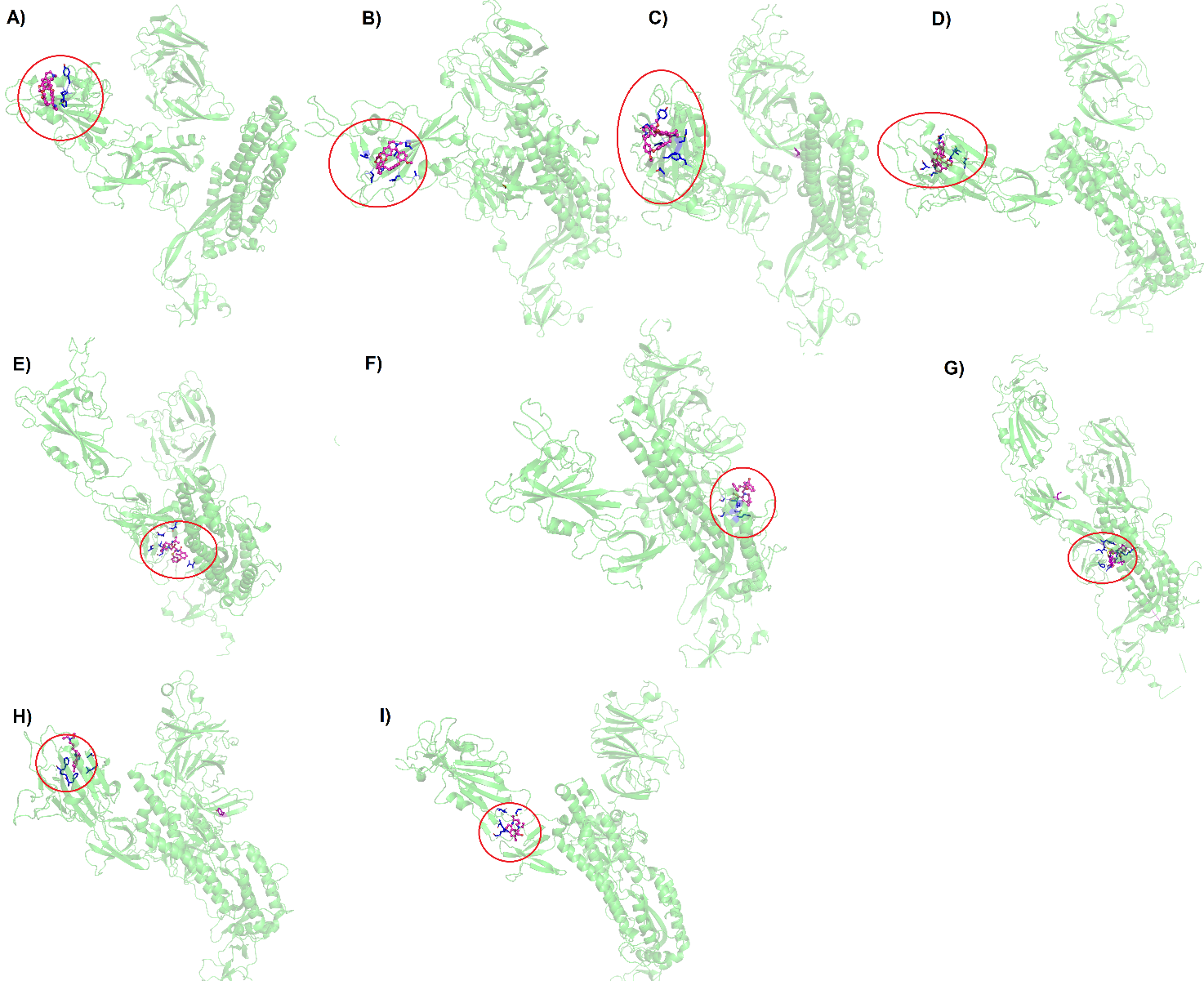


**Figure S3.** Most populated cluster conformation of the ligand-Spike protein complexes retrieved from molecular dynamic simulation using Clustering analysis. Cepharanthine with A) Wild type, B) D614G, C) H49Y, D) T563I; Nelfinavir with E) Wild type, F) H49Y, G) T563I and Hydroxychloroquine with H) H49Y, G) T563I. Ligands are represented as ball-and sticks colored by heteroatoms in magenta, proteins are depicted as green ribbon, and the interacting residues as blue sticks.


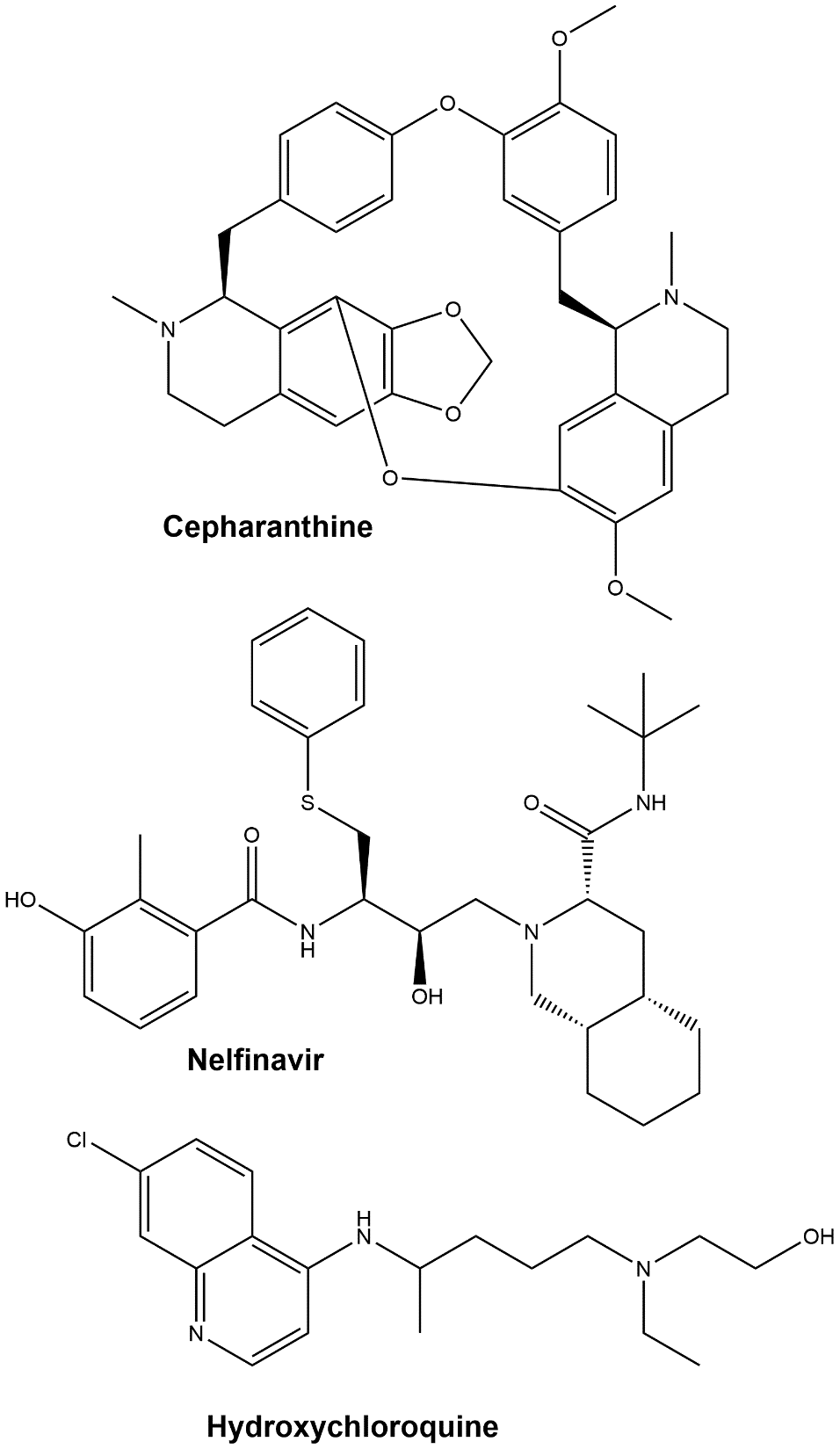


**Figure S4.** 2D structure of the compounds tested against spike protein.

**Table S1**. Sequences of SARS-CoV-2

| **#** | **Type** | **Source** | **ID** |
| --- | --- | --- | --- |
| **1** | hCoV-19 | IPBCAMS-WH-01 | EPI_ISL_402123 |
| **2** | hCoV-19 | CDMX-INCMNSZ_04 | EPI_ISL_426364 |
| **3** | hCoV-19 | CDMX-INCMNSZ_03 | EPI_ISL_426363 |
| **4** | hCoV-19 | CDMX-INCMNSZ_01 | EPI_ISL_426361 |
| **5** | hCoV-19 | CDMX-INCMNSZ_02 | EPI_ISL_426362 |
| **6** | hCoV-19 | CDMX-InDRE_06 | EPI_ISL_424673 |
| **7** | hCoV-19 | Puebla-InDRE_05 | EPI_ISL_424672 |
| **8** | hCoV-19 | Queretaro-InDRE_04 | EPI_ISL_424670 |
| **9** | hCoV-19 | EdoMex-InDRE_03 | EPI_ISL_424667 |
| **10** | hCoV-19 | CDMX-INER_05 | EPI_ISL_424627 |
| **11** | hCoV-19 | CDMX-INER_04 | EPI_ISL_424626 |
| **12** | hCoV-19 | CDMX-INER_01 | EPI_ISL_424345 |
| **13** | hCoV-19 | CDMX-InDRE_01 | EPI_ISL_412972 |
| **14** | hCoV-19 | PUE-InDRE-17 | EPI_ISL_455455 |
| **15** | hCoV-19 | BCN-InDRE-45 | EPI_ISL_516609 |
| **16** | hCoV-19 | CHH-InDRE-43 | EPI_ISL_516613 |
| **17** | hCoV-19 | SON-InDRE_35 | EPI_ISL_516622 |
| **18** | hCoV-19 | AGU-InDRE-55 | EPI_ISL_576258 |
| **19** | hCoV-19 | TLA-InDRE-57 | EPI_ISL_576260 |
| **20** | hCoV-19 | NLE-InDRE-59 | EPI_ISL_576262 |
| **21** | hCoV-19 | OAX-InDRE-61 | EPI_ISL_576264 |
| **22** | hCoV-19 | QRO-InDRE-84 | EPI_ISL_658878 |
| **23** | hCoV-19 | QROO-InDRE-53 | EPI_ISL_658904 |
| **24** | hCoV-19 | SLP-InDRE-77 | EPI_ISL_660068 |
| **25** | hCoV-19 | CMX-INER-0112 | EPI_ISL_837710 |
| **26** | hCoV-19 | CMX-INER-0051 | EPI_ISL_837604 |
| **27** | hCoV-19 | CMX-INER-0160 | EPI_ISL_837756 |
| **28** | hCoV-19 | CMX-INER-0205 | EPI_ISL_843192 |
| **29** | hCoV-19 | CMX-INER-0214 | EPI_ISL_837807 |
| **30** | hCoV-19 | QRO-InDRE-90 | EPI_ISL_658893 |
| **31** | hCoV-19 | AGS-InDRE-89 | EPI_ISL_658891 |
| **32** | hCoV-19 | QRO-InDRE-88 | EPI_ISL_658888 |
| **33** | hCoV-19 | QRO-InDRE-87 | EPI_ISL_658886 |
| **34** | hCoV-19 | AGS-InDRE-86 | EPI_ISL_658883 |
| **35** | hCoV-19 | AGS-InDRE-85 | EPI_ISL_658880 |
| **36** | hCoV-19 | AGS-InDRE-83 | EPI_ISL_658875 |
| **37** | hCoV-19 | AGS-InDRE-82 | EPI_ISL_658873 |
| **38** | hCoV-19 | AGS-InDRE-81 | EPI_ISL_658870 |
| **39** | hCoV-19 | AGS-InDRE-80 | EPI_ISL_658868 |
| **40** | hCoV-19 | AGS-InDRE-79 | EPI_ISL_658865 |
| **41** | hCoV-19 | AGS-InDRE-78 | EPI_ISL_658863 |
| **42** | hCoV-19 | CMX-INER-0222 | EPI_ISL_837814 |
| **43** | hCoV-19 | CMX-INER-0220 | EPI_ISL_837812 |
| **44** | hCoV-19 | CMX-INER-0193 | EPI_ISL_837789 |
| **45** | hCoV-19 | CMX-INER-0191 | EPI_ISL_837787 |
| **46** | hCoV-19 | CMX-INER-0219 | EPI_ISL_837811 |
| **47** | hCoV-19 | CMX-INER-0183 | EPI_ISL_837779 |
| **48** | hCoV-19 | CMX-INER-0172 | EPI_ISL_837768 |
| **49** | hCoV-19 | CMX-INER-0090 | EPI_ISL_837688 |
| **50** | hCoV-19 | CMX-INER-0119 | EPI_ISL_837716 |

**Table S2.** Cluster analysis using RMSD cut-off of 2.0 Å.

|  | **Cluster population (%)** | | | |
| --- | --- | --- | --- | --- |
| **Cluster** | **WT** | **D614G** | **H49Y** | **T531I** |
| **1** | 8.4 | 10.6 | 6.3 | 7.8 |
| **2** | 6.8 | 6.4 | 6.1 | 6.9 |
| **3** | 5.7 | 6.1 | 5.9 | 6.8 |
| **4** | 5.1 | 6.1 | 5.2 | 5.1 |
| **5** | 4.4 | 5.3 | 3.9 | 4.4 |
| **6** | 4.4 | 4.7 | 3.8 | 4.4 |
| **7** | 4.4 | 4.5 | 3.6 | 4.2 |
| **8** | 4.1 | 4.4 | 3.5 | 4 |
| **9** | 3.9 | 4.2 | 3.5 | 3.6 |
| **10** | 3.6 | 3.7 | 3.4 | 3.4 |
| **11** | 3.3 | 3 | 3.3 | 3 |
| **12** | 3.2 | 3 | 3.3 | 3 |
| **13** | 3.1 | 3 | 3 | 2.9 |
| **14** | 2.9 | 2.7 | 2.9 | 2.9 |
| **15** | 2.8 | 2.4 | 2.8 | 2.8 |
| **16** | 2.7 | 2.2 | 2.7 | 2.5 |
| **17** | 2.6 | 2 | 2.6 | 2.5 |
| **18** | 2.4 | 2 | 2.5 | 2.4 |
| **19** | 2.3 | 2 | 2.3 | 2.3 |
| **20** | 2.2 | 1.8 | 2.2 | 2.3 |
| **21** | 2.1 | 1.8 | 2.2 | 2.2 |
| **22** | 2.1 | 1.8 | 2.2 | 2.2 |
| **23** | 2 | 1.7 | 2.1 | 2.2 |
| **24** | 1.9 | 1.7 | 2.1 | 2.1 |
| **25** | 1.9 | 1.7 | 2 | 2.1 |
| **26** | 1.8 | 1.6 | 2 | 1.8 |
| **27** | 1.6 | 1.6 | 2 | 1.6 |
| **28** | 1.4 | 1.6 | 1.9 | 1.5 |
| **29** | 1.4 | 1.4 | 1.8 | 1.5 |
| **30** | 1.4 | 1.1 | 1.8 | 1.3 |
| **31** | 1.2 | 1.1 | 1.7 | 1.3 |
| **32** | 1.1 | 1.1 | 1.5 | 1.2 |
| **33** | 0.9 | 0.9 | 1.5 |  |
| **34** | 0.9 | 0.8 | 1.3 |  |

**Table S3.** Binding free energy of compounds docked with wild type spike protein and mutants.

|  | **Binding free energy (Kcal/mol)** |
| --- | --- |
| **Cepharanthine** |  |
| **D614G** | -5.95 |
| **H49Y** | -6.42 |
| **T563I** | -5.39 |
| **WT** | -6.57 |
| **Hydroxychloroquine** |  |
| **D614G** | -4.55 |
| **H49Y** | -4.66 |
| **T563I** | -4.19 |
| **WT** | -4.46 |
| **Nelfinavir** |  |
| **D614G** | -6.1 |
| **H49Y** | -7.52 |
| **T563I** | -5.38 |
| **WT** | -7.88 |

***Non-Covalent Interaction index (NCI)***

The non-covalent interaction index^1^ is based on the analysis of the reduced density gradient, s(r), which is defined as

$$s \left( r \right)=\frac{|\Delta\rho\left( r \right)|}{{2\left( 2\pi^{2} \right)}^{1/3}\rho\left( r \right)^{4/3}}$$

where ρ(r) is the electron density. This function measures the deviation of the electron density of a system with regards to a uniform electron gas, which is a quantum model in which both the positive charges and the electron density are distributed homogeneously in space. It has been shown that when s(r) is plotted against ρ(r) for molecular complexes some “peaks” are observed at low s(r) and r values, which are absent in the case of the isolated molecules. Therefore, these peaks can be considered as “signatures” of intermolecular interactions. For example, the plots of s(r) as a function of r for the methane and methane dimer are depicted in Figure S5. As can be seen, a peak appears in the case of the dimer which can be interpreted as evidence of intermolecular interactions. These 2D plots are known as NCI diagrams. As well, a 3D representation of the regions where intermolecular interactions are present can be obtained from isosurfaces of  s(r). Furthermore, the strength and classification of the type of non-covalent interaction can be obtained by the analysis of the function sign(λ_2_)*r, where sign(λ_2_) is the sign of the second eigenvalue of the electron density Hessian matrix. In cases where λ_2_ > 0 the electron density is depleted, which is correlated with steric repulsion. On the other hand, when λ_2_ < 0 the electron density is accumulated, as will be expected for strong interactions such as hydrogen bond. Finally, regions where r 0 are typical of weak interactions (van der Waals forces), irrespective of sign(λ_2_). Taking this into account, the 3D NCI isosurfaces are constructed to identify the regions and type of non-covalent interactions in real space. For instance, the NCI isosurface of the methane dimer is depicted in Figure S5. A green flat surface is observed, which corresponds to the weak van der Waals forces between the two molecules. The NCI isosurface for the phenol dimer is depicted in Figure S6. The three type of interactions are observed in this case: a blue small disk, where λ_2_ < 0 and is associated with the strong O-H•••H hydrogen bond formed between the two molecules; a red surface in the middle of the aromatic rings, where λ_2_ > 0 and is correlated with the steric clash of the ring structure; and flat surface between the aromatic rings, where r 0 and represent the π•••π interactions.

Lastly, a quantitative analysis can be obtained from the integration of r or some of its powers in the NCI isosurfaces. The integration of the electron density has been shown to correlate with interaction energies^2^. Moreover, r can be integrated with a different sign(λ_2_)*r intervals to study the contribution of each type of non-covalent interactions. A typical example would be to integrate in the following intervals (given in atomic units): from –0.1 to -0.02 for strong attractive interactions, from -0.02 to 0.02 for weak interactions, and from 0.02 to 0.1 for repulsive interactions.


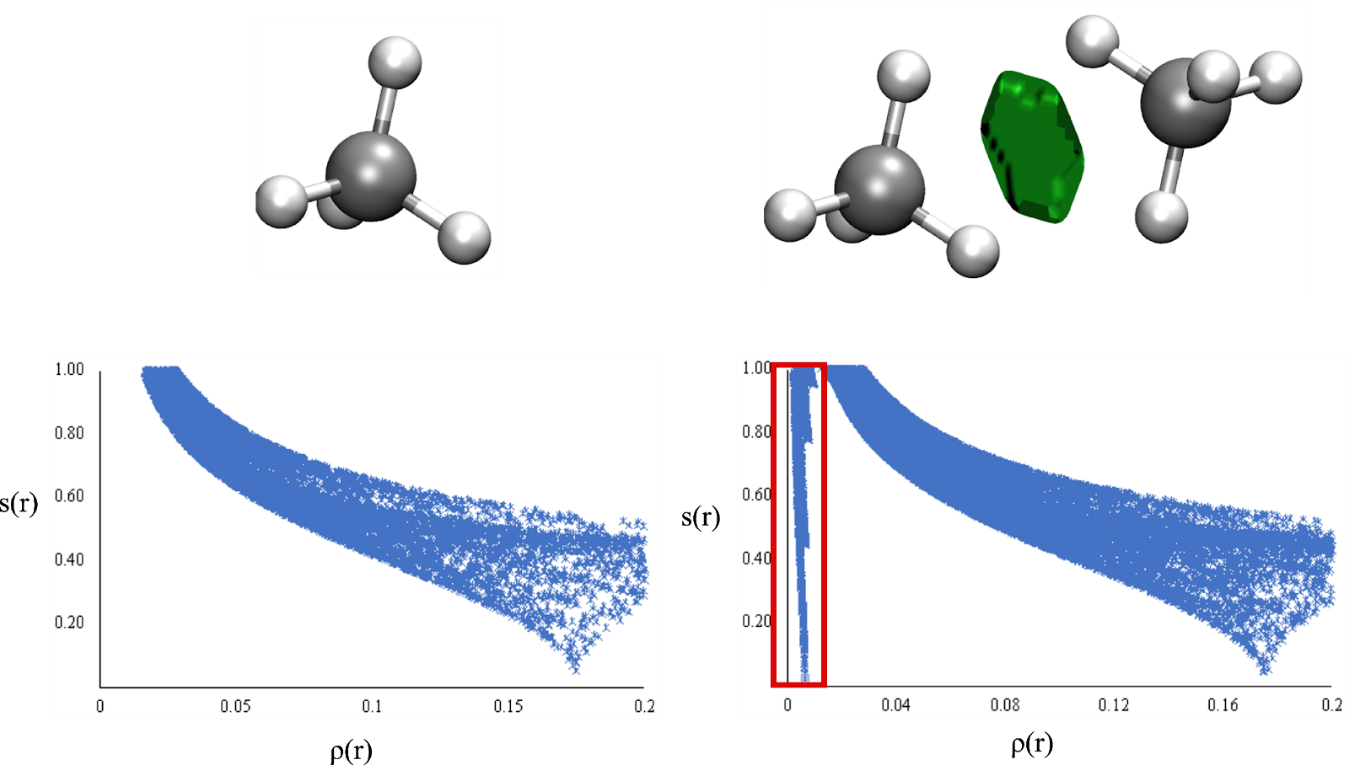


**Figure S5**. Top: NCI models of methane and methane dimer. In the later, the NCI isosurface is depicted, where a green flat surface is observed and corresponds to weak van der Waals interactions between the two molecules. Bottom, NCI diagrams of methane and methane dimer. The peak corresponding to the non-covalent interactions is marked with a red rectangle in the case of the dimer.


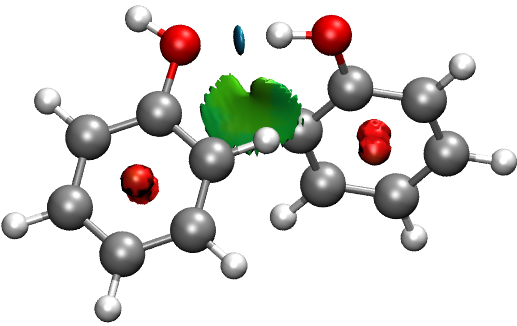


**Figure S6**. NCI isosurfaces of the phenol dimer.

 Since the computation of electron densities from quantum mechanical calculations is expensive for biological systems, promolecular densities (those derived from the superposition of individual atomic densities) can be used instead. It has been demonstrated that similar results are obtained from both types of electron density models^1^.

**References**

1. Johnson, E. R. *et al.* Revealing Noncovalent Interactions. *J. Am. Chem. Soc.* **132**, 6498–6506 (2010).

2. Peccati, F. NCIPLOT4 Guide for Biomolecules: An Analysis Tool for Noncovalent Interactions. *J. Chem. Inf. Model.* **60**, 6–10 (2020).
